# Chemical priming of plant defense responses to pathogen attacks

**DOI:** 10.3389/fpls.2023.1146577

**Published:** 2023-05-08

**Authors:** Martin Hönig, Venja M. Roeber, Thomas Schmülling, Anne Cortleven

**Affiliations:** ^1^ Institute of Biology/Applied Genetics, Dahlem Centre of Plant Sciences (DCPS), Freie Universität Berlin, Berlin, Germany; ^2^ Department of Chemical Biology, Faculty of Science, Palacký University, Olomouc, Czechia

**Keywords:** *Arabidopsis thaliana*, biotic stress, chemical priming, defense priming, induced systemic resistance, pathogen attack, priming, systemic acquired resistance

## Abstract

Plants can acquire an improved resistance against pathogen attacks by exogenous application of natural or artificial compounds. In a process called chemical priming, application of these compounds causes earlier, faster and/or stronger responses to pathogen attacks. The primed defense may persist over a stress-free time (lag phase) and may be expressed also in plant organs that have not been directly treated with the compound. This review summarizes the current knowledge on the signaling pathways involved in chemical priming of plant defense responses to pathogen attacks. Chemical priming in induced systemic resistance (ISR) and systemic acquired resistance (SAR) is highlighted. The roles of the transcriptional coactivator NONEXPRESSOR OF PR1 (NPR1), a key regulator of plant immunity, induced resistance (IR) and salicylic acid signaling during chemical priming are underlined. Finally, we consider the potential usage of chemical priming to enhance plant resistance to pathogens in agriculture.

## 1 Introduction

In natural and agricultural habitats, plants must cope with constantly changing environmental conditions. Some of these conditions have the potential to severely stress plants; therefore, sensing and responding to specific environmental stimuli is important to ensure their survival. Interestingly, plants do not only directly respond to environmental conditions but also remember previous stimuli over a stress-free time (lag phase). The memory of previous stimuli can prepare or “prime” plant responses to stresses occurring in the future, thereby triggering earlier, faster and/or stronger responses (Conrath et al., 2002; Hilker et al., 2016; Mauch-Mani et al., 2017; Hilker and Schmülling, 2019). Several stimuli have been shown to prime plants for an improved response to future stress. Probably the best-known examples of priming involve the plant immune system, which is activated by a first pathogen infection establishing an immune memory that improves the plants’ resistance to second infection (Hake and Romeis, 2019).

Previous reviews have mainly focused on chemical priming of abiotic stress responses in plants (Antoniou et al., 2016; Savvides et al., 2016; Kerchev et al., 2020; Rhaman et al., 2021; Sako et al., 2021). Other reviews highlight the chemical priming of seeds (Lutts et al., 2016; Pawar and Laware, 2018; Devika et al., 2021) resulting in improved and/or more reliable germination, or give a general summary of defense priming, including priming to herbivores (Bagheri and Fathipour, 2021), which is not part of this review. In González-Bosch (2018), the possibilities of chemical priming by natural compounds to contribute to a more sustainable agriculture is reviewed. Here, we consider the chemical priming effects against pathogen infections of several compounds and describe the underlying molecular mechanisms and cellular pathways.

### 1.1 The plant immune system

To defend against pathogens, plants developed numerous sensing and signaling mechanisms, including a two-layered innate immune system, which consists of pattern-triggered immunity (PTI) and effector-triggered immunity (ETI). PTI is triggered by conserved microbial patterns (pathogen-associated molecular patterns, PAMPs), which are detected *via* PATTERN RECOGNITION RECEPTORS (PRRs). Pathogen-secreted effector proteins activate the plants’ ETI when recognized *via* nucleotide-binding domain leucine-rich repeat (NLR) receptors, which are predominantly localized inside the plant cell (Jones and Dangl, 2006; Pruitt et al., 2021). Effective defense against pathogens is ensured by the mutual potentiation of plant immunity by cell-surface and intracellular receptors (Ngou et al., 2021; Yuan et al., 2021). Plants can further induce their basal resistance upon appropriate stimulation (by e.g. pathogens, herbivores, chemicals) in a process referred to as induced resistance (IR) (Bagheri and Fathipour, 2021), which decreases the plants’ susceptibility to future challenges (De Kesel et al., 2021). IR phenotypes result from either a local and/or a systemic establishment of plant defense responses. Local resistance is observed only in those parts of the plants that are initially subjected to the IR stimulus, while systemic induced resistance (ISR) improves the resistance in the entire plant. In addition, IR phenotypes result from either direct or induced/primed defense responses. Priming responses are characterized by a potentiation of defense responses upon pathogen attacks (leading to earlier, faster and/or stronger defense responses) and are not activated if the pathogen attack is lacking (De Kesel et al., 2021). The characteristics of IR phenotypes are not mutually exclusive meaning that the final defense responses result from a combination of direct and primed responses. Similarly, a strong local response does not exclude systemic effects (De Kesel et al., 2021).

From the chemical perspective, IR is primarily orchestrated by three molecules, namely the phytohormones salicylic acid (SA), jasmonic acid (JA) and ethylene (ET), which are interconnected by complex signaling pathway networks to fine-tune the plant defense (Pieterse et al., 2009).

### 1.2 Chemical priming of plant defense responses to pathogen attacks

Plants can acquire an improved resistance to pathogen attacks by exogenous application of natural and artificial (non-organismal) compounds (Conrath, 2009; Zhou and Wang, 2018). This chemical priming causes an enhanced resistance involving earlier, faster and/or stronger responses upon pathogen attacks and is also often observed after a stress-free time (lag phase) or in plant organs that have not been directly treated with the compounds (Hilker and Schmülling, 2019).

## 2 Naturally occurring priming processes

In the following sections, we will give a general introduction to naturally occurring priming concepts exemplified by priming responses of systemic acquired resistance (SAR) (section 2.1), and priming of ISR by beneficial microorganisms (section 2.2).

### 2.1 Priming of SAR

Priming of SAR, which is activated upon a local infection and, enables resistance in systemic plant tissues, represents one of the best described specific type of ISR (Klessig et al., 2018; Zhou and Zhang, 2020). SA and *N*-hydroxypipecolic acid (NHP) are the two major chemical signals that control SAR in *Arabidopsis thaliana* (Gaffney et al., 1993; Chen et al., 2018; Hartmann et al., 2018; Hartmann and Zeier, 2019). The establishment of SAR *via* SA and/or NHP is dependent on the transcriptional co-activator NONEXPRESSOR OF PATHOGENESIS-RELATED GENES 1 (NPR1), which enables the characteristic expression of pathogenesis-related (*PR*) protein genes with antimicrobial functions (Cao et al., 1998; van Loon et al., 2006; Backer et al., 2019). Transcriptional responses are further regulated by the repressors NPR1-LIKE PROTEIN3 (NPR3) and NPR4. As SA levels increase during pathogen infections, SA binds to NPR3/NPR4 causing their transcriptional repression of defense genes (Ding et al., 2018). Apart from their action as transcriptional corepressors, NPR3/NPR4 regulate SA-dependent degradation of NPR1, which is realized through their function as adaptors of the cullin3 ubiquitin E3 ligase (Fu et al., 2012; Wang et al., 2020). Recently, it was found that perception of SA by NPR1 and NPR4 is also required for activation of NHP biosynthesis, which serves as a mobile signal and is essential for SAR induction (Liu et al., 2020). SA itself can also serve as a mobile chemical signal transported to uninfected tissues to prepare them for subsequent stress, preferably through the apoplast (Lim et al., 2016; Lim et al., 2020). In addition, SA can regulate plasmodesmata gating and thereby transport of other mobile signals such as azelaic acid (AZA) or glycerol-3-phosphate (G3P) (Kachroo and Kachroo, 2020).

Chemical priming (of SAR) can be achieved *via* direct application of SA and NHP (Kauss et al., 1992b; Yi et al., 2014; Yi et al., 2015; Hartmann and Zeier, 2019; Yildiz et al., 2021), application of their precursors (Bernsdorff et al., 2016; Hartmann et al., 2018), or induction of their biosynthesis pathways (Návarová et al., 2012; Hartmann et al., 2018; Koley et al., 2022). The mobilization of SA and NHP is important in the process of chemical priming. Downstream of SA and NHP, NPR1 plays an essential role in orchestrating SAR (Kohler et al., 2002; Backer et al., 2019; Yildiz et al., 2021). SA directly binds to the transcriptional coregulator NPR1 to induce SAR gene expression, which leads to enhanced plant defense (Wu et al., 2012). Notably, activation of NPR1 through SA is dependent on the plant redox state (Saleem et al., 2021). The perception of NHP still needs to be elucidated; as yet, no receptor has been identified (Hartmann and Zeier, 2019; Guerra and Romeis, 2020). It was shown that despite structural similarities, NHP does not bind to NPR1 *in vitro*, while SA binds with a Kd of 585 ± 368 nM (Nair, 2021). Nevertheless, NHP primes plants involving SAR in both a SA-dependent and, to a lesser degree, a SA-independent manner (Bartsch et al., 2006; Mishina and Zeier, 2006; Bernsdorff et al., 2016).

Priming within SAR was also studied using artificial compounds, namely the SA structural analogue benzothiadiazole (BTH) (Conrath et al., 2002). BTH activity is dependent on NPR1 (Kohler et al., 2002), which even has a slightly higher affinity for BTH than for SA (Wu et al., 2012). Interestingly, BTH inhibits the activity of the antioxidant enzyme catalase stronger than SA (Wendehenne et al., 1998). Similar as SA (Yi et al., 2015), BTH treatment of *Arabidopsis* resulted in priming associated with the accumulation of mRNAs and inactive proteins of mitogen-activated protein kinase3 (MPK3) and MPK6 (Beckers et al., 2009). Plants primed through SAR equip themselves with pattern recognition receptors (Tateda et al., 2014; Hartmann et al., 2018) and pathogen-responsive MPKs (Beckers et al., 2009) which are then activated upon a second infection by elicitors such as the flagellin-derived peptide flg22 and depend on NPR1 (Yi et al., 2015).

Along with the accumulation of inactive signaling molecules, chemicals prime plants through chromatin modifications. In parsley suspension cultures, BTH application caused covalent modifications of histone H3 and chromatin opening in the *WRKY6* and *PR1* regulatory regions and enhanced *WRKY6* activation upon flg22 treatment (Schillheim et al., 2018). In *Arabidopsis* seedlings, BTH treatment primes plants for augmented expression of *WRKY* transcription factor genes, which are involved in the regulation of plant stress, namely *WRKY6*, *WRKY29* and *WRKY53*. This gene priming is associated with chromatin modifications on *WRKY* gene promoters, especially H3K4 trimethylation (H3K4me3). Application of BTH alone, however, does not activate the expression of *WRKY6*, *WRKY29* and *WRKY53*. Enhanced gene activation is only observed subsequently after a stress stimulus. Notably, neither augmented *WRKY* gene expression nor high induction of H3K4 trimethylation was observed in *npr1* mutants in response to priming (by BTH) (Jaskiewicz et al., 2011), showing again the crucial role of NPR1 in priming for SAR.

In conclusion, chemical priming agents using SAR were shown to increase transcription of pattern recognition receptors genes and accumulate MPK3 and MPK6 in the form of the corresponding mRNAs and inactive proteins that can be activated during the following stress. Additionally, epigenetic changes allowed fast activation of stress response-related transcription factors. In this way, plants are able to enhance and fasten the response to pathogens. Primed plants then responded to stress with elevated expression of pathogen-responsive genes (Mur et al., 1996; Návarová et al., 2012; Koley et al., 2022), phytoalexin production (Kauss et al., 1992b; Thulke and Conrath, 1998; Návarová et al., 2012; Vogel-Adghough et al., 2013; Akagi et al., 2014; Hartmann et al., 2018; Yildiz et al., 2021) and/or an enhanced oxidative burst (Kauss and Jeblick, 1995).

### 2.2 Priming of ISR triggered by beneficial microbes

JA and ET, but also a synergism of JA and SA signaling pathways (Vlot et al., 2021), play an important role in the interaction of plants with beneficial plant growth-promoting rhizobacteria (PGPR) or fungi (PGPF). Interaction of PGPR/F with plants causes ISR, which primes the plants’ defense against JA- and ET-sensitive necrotrophic pathogens (Conrath et al., 2002; Pieterse et al., 2014) and additionally also improves the plants’ resistance in aerial tissues against (hemi-) biotrophic pathogens (Vlot et al., 2021). Recent data suggest that the interplay between different phytohormone signaling pathways determines the outcome of ISR and this depends on the plant, the microbial resistance inducer, and the phytopathogen (Vlot et al., 2021).

In *Arabidopsis*, ISR by PGPR is not associated with a direct effect on the expression of defense-related genes or hormone biosynthesis but rather with the potentiation of the expression of specific JA- and ET-responsive genes (van Wees et al., 1999; Pieterse et al., 2000; Pieterse et al., 2014). In addition, ISR is not triggered in JA- and ET-insensitive mutants (Pieterse et al., 2000) pointing to an essential role of JA and ET in this process. The APETALA2/ETHYLENE RESPONSIVE FACTOR (AP2/ERF) transcription factor family is associated with both JA- and ET-regulated plant defense and priming *via* ISR (van der Ent et al., 2009; Pieterse et al., 2014). Concurrently, JA- and ET-regulated genes showed an augmented expression in ISR-primed *Arabidopsis* after *Psm* infection (Verhagen et al., 2004). *A. thaliana* plants growing with ISR-inducing *P. fluorescens* WCS417r bacteria showed an increased expression of JA-responsive genes in comparison to plants growing without the bacteria. The G-box-like motif binding site for a key transcription factor of JA-mediated defense, JASMONATE INSENSITIVE1 (MYC2), was found to be significantly enriched among JA-responsive genes in ISR-primed *Arabidopsis*. Moreover, *jin1-1* and *jin1-2*, which are mutated in the *JASMONATE-INSENSITIVE1/MYC2* gene, were not primed *via* ISR (Pozo et al., 2008), showing the central role of MYC2 as a transcriptional regulator in this process (Panpatte et al., 2020). The importance of JA- and ET-responsiveness was also shown by treatment of *Arabidopsis* plants with either methyl jasmonate (MeJA) or the ethylene precursor 1-aminocyclopropane-1-carboxylate (ACC), which both induced resistance to *Pst* DC3000 when applied three days prior to bacteria inoculation (Pieterse et al., 1998).

Notably, priming by both PGPR and its signaling molecules failed in *npr1* mutants (Pieterse et al., 1998), showing the crucial role of the transcriptional co-activator NPR1 also in PGPR-based priming. Interestingly, the involvement of NPR1 in ISR by PGPR is not dependent on SA itself (Pieterse et al., 2014).

## 3 Natural chemical priming compounds

In the following sections, we give a summary on naturally occurring priming compounds. We considered SA (section 3.1), NHP and pipecolic acid (Pip, section 3.2), azelaic acid (AZA, section 3.3), methyl jasmonate (MeJA, section 3.4), beta-aminobutyric acid (BABA, section 3.5), gamma-aminobutyric acid (GABA, section 3.6), cytokinins (CKs, section 3.7) and hexanoic acid (Hx, section 3.8).

### 3.1 Salicylic acid

SA is a phytohormone involved in the regulation of various aspects of plant growth and development, but foremost in plant immunity (Vlot et al., 2009). As described above, it is one of the critical regulators of SAR and plant defense against biotrophic and hemibiotrophic pathogens (Glazebrook, 2005) and piercing-sucking herbivores (Bagheri and Fathipour, 2021). SA plays also an important role in regulating growth-defense trade-offs (Rivas-San Vicente and Plasencia, 2011; van Butselaar and van den Ackerveken, 2020; Peng et al., 2021). To optimize defense without unnecessary fitness costs, SA levels are controlled in plants through regulation of biosynthesis as well as metabolism. Moreover, complex feedback loops involving both transcriptional and posttranslational regulation balance SA signaling output (for reviews see Ding and Ding, 2020; Peng et al., 2021).

**Figure 1:**
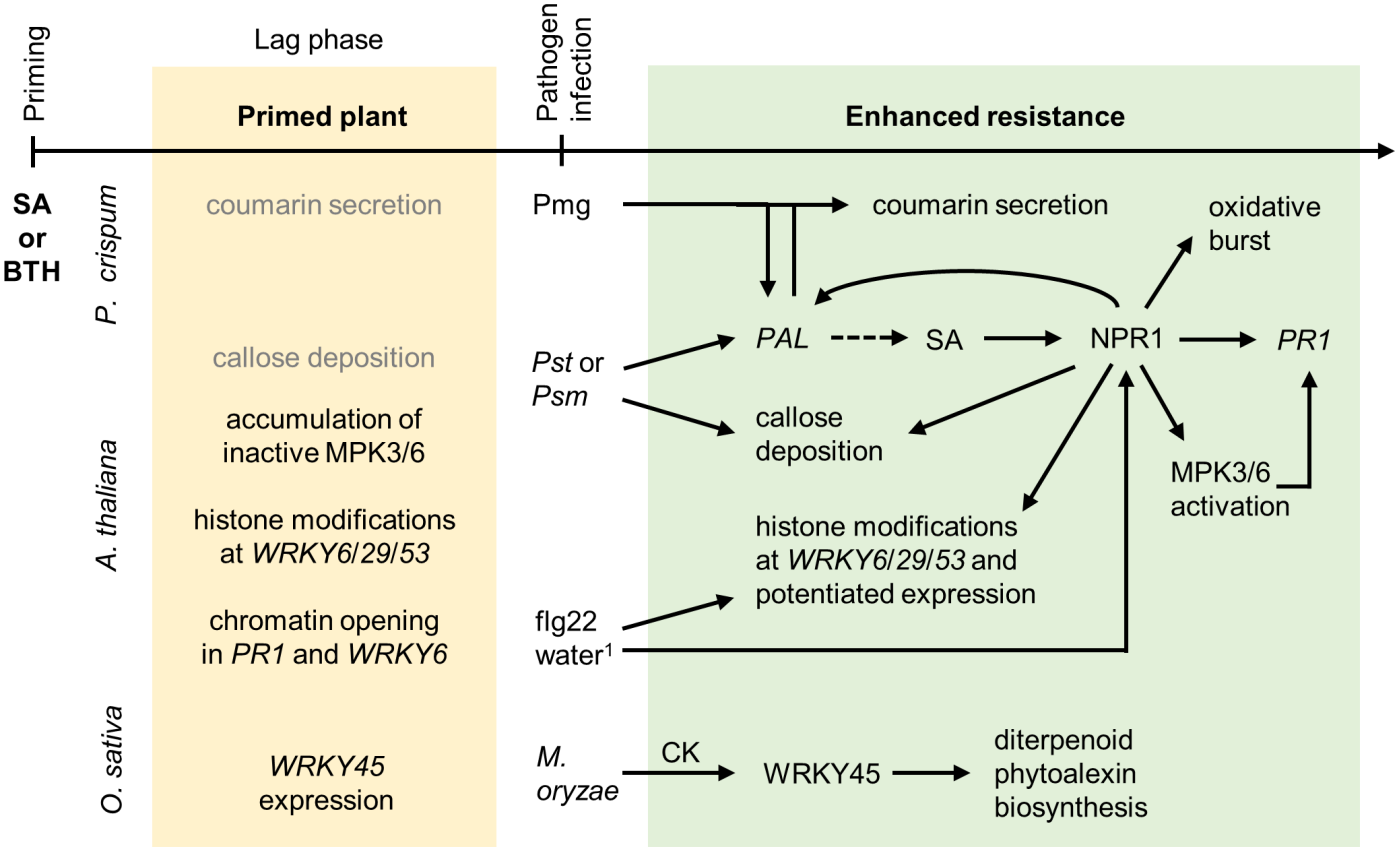
Priming by salicylic acid and benzothiadiazole. Chemical priming by SA and its structural analog BTH is best described in *P. crispum* L. (parsley) suspension culture and *A. thaliana.* Primed plants accumulate small amounts of the phytoalexin coumarin, and the secretion increases strongly after *Pst* infection or elicitor application^1^, which is accompanied by augmented *PHENYLALANINE AMMONIA-LYASE* (*PAL)* expression. PAL induces SA biosynthesis (dashed line arrow) and thus further enhances disease resistance. Chemical treatment has a limited direct effect on callose deposition, which, however, increases rapidly after pathogen infection. Additionally, SA treatment enhances the oxidative burst after the application of a fungal elicitor. Primed plants accumulate inactive MPK3 and MPK6 and their respective mRNAs. After pathogen infection, accumulated MPKs are activated by phosphorylation allowing for stronger immune responses. The primed plant possesses chromatin modifications at the *WRKY* transcription factor and *PR1* regulatory regions. This allows their augmented expression after flg22 application or stress by water infiltration^1^. The transcriptional coregulator NPR1 is the key player in chemical priming required for the majority of the described processes. In *O. sativa* cv. NB, diterpenoid phytoalexin biosynthesis is augmented upon infection by *M. oryzae* in a process dependent on WRKY45 and mediated by CK, especially isopentenyladenine. Light grey color, effects are less pronounced in primed plants than in primed-and-infected plants. *^1^Water infiltration stress causes cell collapse or a wound stress response in the leaf*.

SA as a chemical priming agent has been studied since the early 1990s, and most of the work was done in parsley (*Petroselinum crispum* L.) suspension culture (**Figure 1**). Pretreatment with SA augmented the secretion of the phytoalexin coumarin in parsley cells (Kauss et al., 1992b). SA also potentiated the expression of the SA- and coumarin biosynthetic gene *PHENYLALANINE AMMONIA LYASE* (*PAL*) upon exposure to low doses of cell wall elicitor from *Phytophthora megasperma* f. sp. *glycinea* (*Pmg*) (Thulke and Conrath, 1998). Additionally, SA-pretreated parsley cells showed stronger elicitation of an oxidative burst (Kauss and Jeblick, 1995). The K^+^/pH response was more rapidly induced in SA-primed parsley cells resulting in increased antibacterial coumarin secretion (Katz et al., 2002). Furthermore, exogenously applied SA induced defense gene activation correlated with improved disease resistance in parsley cell cultures (Thulke and Conrath, 1998). Consistently, in soybean (*Glycine max*) cell suspension cultures, physiological concentrations of SA significantly enhanced the induction of defense gene transcripts, H_2_O_2_ accumulation, and hypersensitive cell death by an avirulent strain of *Pseudomonas syringae* pv *glycinea* (*Psg*) (Shirasu et al., 1997). The importance of ROS production for priming by SA was also shown in an experiment with cucumber (*Cucumis sativus* L.) hypocotyls. SA treatment augmented H_2_O_2_ production upon treatment by elicitor from *Phytophora sojae* (Fauth et al., 1996). The effects of SA pretreatment were also studied in tobacco, *Arabidopsis*, tomato and beans. SA pretreatment potentiated the expression of the fusion genes *AoPR1-GUS* and *PAL-GUS* in transgenic tobacco plants after *Pseudomonas syringae* pv. *syringae* (*Pss*) and tobacco mosaic virus (TMV) infection (Mur et al., 1996). In *Arabidopsis*, SA pretreatment enhanced *via* NPR1 acting downstream of SA biosynthetic enzyme SALICYLIC ACID INDUCTION DEFICIENT2 (SID2)/ISOCHORISMATE SYNTHASE1 (ICS1) a flg22-triggered oxidative burst, callose deposition and the induction of the stress marker genes *WRKY29* and *FLG22-INDUCED RECEPTOR-LIKE KINASE1* (*FRK1)* (Yi et al., 2014). In another study, SA pretreatment enhanced dual phosphorylation of the TEY motif in MPK3 and MPK6 upon flg22 treatment. This also required intact NPR1 (Yi et al., 2015), indicating that NPR1 is essential for the SA priming effect. In tomato, Koley et al. (2022) showed that SA primes against infection by the necrotrophic pathogen *Rhizoctonia solani*. SA pretreatment induced biosynthesis of SA, JA and polyphenols, caused a stronger *PR1a* and *PHYTOALEXIN-DEFICIENT4* gene *(PAD4*) induction, limited pathogen-stimulated ROS production upon pathogen infection and also enhanced callose deposition in the early phase of the fungal infection.

Together, these results show that SA possesses a dual role in the activation of plant defense responses (Thulke and Conrath, 1998; Conrath, 2009). It directly activates plant defense genes and potentiates their expression upon pathogen infection. Although the beneficial effects of SA applied exogenously under laboratory conditions have been known for more than three decades and are well studied (**Table 1**), it is not utilized as priming agent in agriculture. Field experiments showed that SA is insufficiently tolerated by some crop plants to warrant its practical use as plant protection compound (Ryals et al., 1996).

**Table 1:** Chemical priming of plant defense responses to pathogens.

	Plant species	App^1^	Pathogens	Lag^2^	Priming effect	References
**SA**	*P. crispum* L. sc.	Medium^3^	Pmg	1 d^4^	Increased coumarin production upon low-dose elicitation.	Kauss et al., 1992b
	*P. crispum* L. sc.	Medium^3^	Pmg	1 d^4^	Enhanced elicitation of the oxidative burst.	Kauss and Jeblick, 1995
	*P. crispum* L. sc	Medium^3^	Pmg	22 h^4^	Potentiated expression of *PAL, 4-coumarate:CoA ligase* (*4CL*), *PR10* and *hydroxyproline-rich glycoprotein* (*HRGP*) upon elicitor treatment.	Thulke and Conrath, 1998; Conrath et al., 2006
	*Glycine max* sc.	Medium^3^	*Psg*	NL^11^	Increased defense gene expression, H_2_O_2_ accumulation, and hypersensitive cell death upon *Psg* infection.	Shirasu et al., 1997
	*P. crispum* L. sc	Medium^3^	Pep-13	1 d^4^	Enhanced induction of a rapid K^+^/pH response.	Katz et al., 2002
	*Cucumis sativus L.* hypocotyl	–	*P. sojae* elicitor	18 h	Augmented H_2_O_2_ production upon treatment by elicitor from *Phytophora sojae.*	Fauth et al., 1996
	*N. tabacum*	Hydroponic	*Pss* 2774 TMV U1	7 d^5^	Potentiation of defense genes *AoPR1-GUS* and *PAL-GUS* upon pathogen attack.	Mur et al., 1996
	*A. thaliana*	Medium^3^	flg22	1 d^4^	Improvement of flg22-induced oxidative burst and callose deposition, requiring NPR1 downstream of SID2.	Yi et al., 2014
	*A. thaliana*	–	flg22	1 d^4^	Enhancement of dual phosphorylation of the TEY motif in MPK3 and MPK6 upon flg22 treatment, which requires NPR1.	Yi et al., 2015
	*Pusa Ruby* variety	Spraying	*Rhizoctonia solani*	1 d	Induction of biosynthesis of SA, JA and polyphenols, stronger *PR1a* and *PAD4* induction and limited pathogen-stimulated ROS production upon pathogen attack.	Koley et al., 2022
**BTH**	*P. crispum* L. sc.	Medium^3^	Pmg	1 d^4^	Potentiated activation of *PAL* gene and enhanced coumarin secretion after elicitor application.	Katz et al., 1998
	*Vigna unguiculata*	Seed soaking	C. destru-ctivum	7 d	Potentiation of PAL and CHI enzymes activity and kievitone and phaseollidin isoflavonoid phytoalexins accumulation after pathogen inoculation.	Latunde-Dada and Lucas, 2001
	*A. thaliana*	Spraying	*Pst* DC3000	3 d	*PAL* gene activation and callose deposition after bacteria treatment requiring NPR1.	Kohler et al., 2002
	*A. thaliana*	Spraying	*Pst* DC3000	3 d	Enhanced plant defense upon pathogen exposure. Full priming requires both MPK3 and MPK6.	Beckers et al., 2009
	*A. thaliana*	Medium^3^	flg22	1 d^4^	Covalent modification of histone H3 and chromatin opening in the *WRKY6* and *PR1* regulatory regions and enhanced *WRKY6* activation upon flg22 treatment.	Schillheim et al., 2018
	*Oryza sativa* cv. NB	Soil^6^	*Magnaporthe oryzae*	1 d	Increased diterpenoid biosynthesis upon infection by *M. oryzae* through SA/CK synergism in a WRKY45-dependent manner.	Akagi et al., 2014
**INA**	*P. crispum* L. sc	Medium^3^	Pmg		Potentiation of coumarin production upon low-dose elicitation.	Kauss et al., 1992b
	*Cucumis sativus L.* hypocotyl	–	*P. sojae* elicitor	18 h	Augmented H_2_O_2_ production upon treatment by elicitor from *Phytophora sojae.*	Fauth et al., 1996
	*Phaseolus vulagris* L.	Infiltration	*Pph*	7 d	Potentiation of induction of *WRKY29* and *WRKY53* gene expression upon pathogen exposure.	Martínez-Aguilar et al., 2016
	*P. vulgaris* L. cv. *Riñón*	Spraying	*Pph* or flg22	7 d	Improvement of plant defense upon pathogen exposure by cell wall remodeling.	De la Rubia et al., 2021
**Pip**	*A. thaliana*	Soil^6^	*Psm* ES4326	1 d	Positive regulation of SA biosynthesis and strong potentiation of camalexin production, pathogen-triggered expression of *ALD1*, *FMO1* and *PR1*.	Návarová et al., 2012
	*N. tabacum*	Soil^6^	*Pstb 6605* *Psm* ES4326	1 d	Rapid and strong accumulation of SA and nicotine following bacterial infection.	Vogel-Adghough et al., 2013
	*A. thaliana*	Soil^6^	*Psm* ES4326 *Psm* lux *Hpa* Noco2	1 d	Orchestration of SA-dependent and partially independent SAR transcriptional response, which depends on FMO1.	Bernsdorff et al., 2016; Hartmann et al., 2018
**NHP**	*A. thaliana*	Soil^6^	*Psm* ES4326 *Psm* lux *Hpa* Noco2	1 d	Improvement of pathogen-triggered activation of defense metabolism, including biosynthesis of SA, Pip, branched-chain amino acids and camalexin. Advanced SA- and pathogen-induced expression of defense-related genes requiring NPR1.	Hartmann et al., 2018; Yildiz et al., 2021
	*A. thaliana*	Leaf inf^7^	*Psm* ES4326	1 d	NHP moves systemically in *Arabidopsis* and rescues the SAR-deficiency of *fmo1* mutants. Changes in SAR gene expression and enhancement of the hypersensitive response causing resistance to bacterial pathogens.	Chen et al., 2018
**AZA**	*A. thaliana*	Leaf inf^7^	*Psm ES4326*	12 h - 48 h	SA accumulation and enhanced *PR1* expression in both local and systemic leaves. Priming in systemic leaves is dependent on the *AZI1* gene.	Jung et al., 2009
	*A. thaliana*	Leaf inf^7^	*Psm ES4326*	2 d	Lipid transfer protein (LTP)-like AZI1 and its paralog EARLI1 are necessary for priming by AZA as shown by PR1 and LOX2 protein induction.	Cecchini et al., 2015
**BABA**	*A. thaliana*	–	*Pst* DC3000	1 d	*PR1* gene expression is enhanced upon pathogen attack.	Zimmerli et al., 2000
	*A. thaliana*	–	*P. parasitica*	1 d	Induction of resistance by callose deposition, hypersensitive response and necrosis formation. Resistance does not require SA, JA, ET or SAR signaling pathways.	Zimmerli et al., 2000
	*A. thaliana*	–	*Pst* DC3000	2 d	Induction of SA-dependent defense responses leading to enhanced *PR1* gene expression and necrosis formation.	Ton et al., 2005
	*A. thaliana*	–	*Hpa*	2 d	Induction of SA-dependent defense responses leading to enhanced necrosis. Priming of phosphatidylinositol- and ABA-dependent defense responses resulting in increased callose deposition.	Ton et al., 2005
	*A. thaliana*	Soil^6^	*Pst* DC3000	2 d	Enhancement of resistance to pathogen exposure. Potentiation of induction of *PR1* expression upon pathogen exposure. *L*-glutamine treatment inhibits BABA-induced resistance.	Wu et al., 2010
	*A. thaliana*	Soil^6^	*Pst* DC3000	2 d	Faster and stronger induction of SA signaling genes upon pathogen attack.	Slaughter et al., 2012
	*A. thaliana*	Soil^6^	*Pst* DC3000	1 - 2 d	Priming by the production of amino acids, IAA, SA and SA-glucosides, and xanthosine. BABA boosts plant primary metabolism by induction of tricarboxylic acids and potentiates phenylpropanoid biosynthesis and the octodecanoic pathway.	Pastor et al., 2014
	*A. thaliana*	Soil^6^ (for 6 d), Rep^8^	*Pst* DC3000 *luxCDABE* or *Hpa* or SA	1, 8, 15 and 21 d	Induction of short-term resistance, which is independent of NPR1 and long-term resistance, which depends on NPR1 and involves priming of SA-regulated defense genes. The histone methyltransferase SUVH4/KRYPTONITE (KYP) is required for maintaining the primed state.	Luna et al., 2014a
	*A. thaliana*	Soil^6^	*Hpa* strain WACO9 or CALA-2	2 d	The interaction of BABA with IMPAIRED IN BABA-INDUCED IMMUNITY1 (IBI1) primes pathogen defense responses.	Luna et al., 2014b
	*A. thaliana*	Soil^6^	*Hpa* strain WACO9	2 d	Binding of BABA to endoplasmic reticulum-located IBI1 primes the translocation of IBI1 to the cytosol. Cytosolic IBI1 interacts with VOZ1/2 transcription factors, thereby suppressing pathogen-induced ABA signaling.	Schwarzenbacher et al., 2020
	*Vitis vinifera*	–	*P. viticola*	1 d	Induction of resistance involving callose deposition, phenylpropanoid-dependent defense mechanisms and JA signaling. BABA pre-treatment potentiates the expression of SA and JA signaling genes upon pathogen exposure.	Hamiduzzaman et al., 2005
	*S. tuberosum* cv. Desiree	Spray^9^	*P. infestans*	2 d	Induction of resistance involving PR protein accumulation.	Bengtsson et al., 2014
	*S. lycopersicum*	Soil^6^	*B. cinerea*	5 d	Induction of plant resistance.	Luna et al., 2016
	*Phaseolus vulgaris* L.	Soil^6^	*Pph*	7 d	Priming of plant defense upon pathogen exposure. Potentiation of induction of *PR1*, *PR4*, *NPR1*, *WRKY6*, *WRKY29* and *WRKY53* gene expression upon pathogen exposure, while BABA treatment itself is ineffective modification of chromatin marks.	Martínez-Aguilar et al., 2016
	*L. sativa* romaine cv. Parris island	Soil^6^	*S. enterica* serovar *typhimurium*	1 d	Induction of resistance by enhanced expression of *PR1* upon pathogen attack.	Chalupowicz et al., 2021
	*N. tabacum* L.	Spray^9^ (for 3 d)	*P. parasitica*	3 d	Hydrogen peroxide accumulation results in the activation of plant defense. Enhancement of callose deposition, production of SA and JA-Ile and expression of SA-, JA- and ET signaling genes upon pathogen exposure.	Ren et al., 2022
	*S. lycopersicum* L. cv. Money Maker	Root^10^ (for 7 d) Rep^8^	*B. cinerea*	10 d	Priming of both SA- and JA-dependent resistance. BABA treatment results in genome-wide DNA methylation.	Catoni et al., 2022
***t*Z**	*A. thaliana*	Spray^9^	*Pst* DC3000	1 d	Potentiation of the activation of SA defense-related gene *PR1* upon bacteria inoculation.	Choi et al., 2010
	*N. tabacum* leaves	Petiole feeding	*Pstb*	1 d^12^	Enhanced resistance against bacteria; mode of action not studied	Großkinsky et al., 2011
**KIN**	*N. tabacum* leaves	Petiole feeding	*Pstb*	1 d^12^	A high phytoalexin-pathogen ratio in the early phase of infection efficiently restricted pathogen growth.	Großkinsky et al., 2011
**BAP**	*N. tabacum* leaves	Petiole feeding	*Pstb*	1 d^12^	Enhanced resistance against bacteria; mode of action not studied.	Großkinsky et al., 2011
	*A. thaliana*	Spray^9^	*Hpa* Noco2	2 d	Enhanced resistance against *Hpa* in a dose‐dependent manner. SA-responsive defense genes were up-regulated in response to BAP pretreatment and inoculation with *Hpa* Noco2.	Argueso et al., 2012
**MeJA**	*Petroselinum crispum* L. suspension culture	Medium^3^	Pmg	1 d^4^	Pretreatment augmented secretion of coumarin derivatives and incorporation of esterified hydroxycinnamic acids and “lignin-like” polymers into the cell wall after elicitor treatment.	Kauss et al., 1992a
	*A. thaliana*	Dipping	*Pst* DC3000	3 d	Priming was impaired in JA response mutant *jar1*, the ET response mutant *etr1* and dependent on NPR1.	Pieterse et al., 1998
	*Phaseolus vulgaris* L.	Spray^9^	*Sclerotinia sclerotiorum* (Lib.) de Bary	12 h	Pretreatment-induced systemic upregulation of *PvChit1*/*PR3* (chitinase), *PvCallose* (callose synthase), *PvNBS-LRR* (NBS-LRR resistance-like protein), and *PvF-box* (F-box family protein-like) genes after pathogen infection.	Oliveira et al., 2015
	Tomato *Pusa Ruby* variety	Spray^9^	*Rhizoctonia solani*	1 d	Higher resistance to pathogen attack. Induction of biosynthesis of SA, JA and polyphenols. Stronger *PR1a* and *PAD4* expression induction and reduction of pathogen-stimulated ROS production. Weaker and slower effect than SA in the same experiment.	Koley et al., 2022
**Hx**	*S. lycopersicum* cv. Ailsa Craig	Hydroponic conditions	*B. cinerea*	2 d	Negative effect on fungal membrane permeability. Improvement of plants’ resistance either if the compound stays on the plant or if it is washed away before pathogen exposure indicating priming.	Leyva et al., 2008
	*S. lycopersicum* Mill (wild-type Ailsa Craig, Rheinlands Ruhm, Moneymaker, and Castlemart)	Root	*B. cinerea* or *P. syringae*	2 d	Induction of resistance to different pathogens. The Hx-induced resistance is not SA-dependent. Induction of ABA-dependent callose deposition. In addition, activation of JA-dependent defense responses involving priming of 12-oxo-phytodienoic acid (OPDA) and JA-isoleucine accumulation.	Vicedo et al., 2009
	*S*. *lycopersicum* Mill. cv. Ailsa Craig	Hydroponic conditions or soil^6^	*Pst* DC3000 or *P. syringae* strain *cmaA* lacking coronatine	2 - 3 d	Counteraction of the negative effects of JA-Ile and coronatine on SA signaling. The JA-precursor OPDA accumulates in Hx-treated plants upon infection. Accumulation of transcripts, such as *LoxD* and *OPR3*, which are involved in OPDA- and JA biosynthesis. Potentiation of the expression of SA signaling genes, such as *PR1* and *PR5*, upon pathogen infection, while the expression of ABA- and ET-related genes is not affected. Inhibition of stomatal re-opening mediated by coronatine, thereby probably preventing the entry of the bacteria to the plant mesophyll.	Scalschi et al., 2013
	*S. lycopersicum* cv. Ailsa Craig	Root	*B. cinerea*	2 d	Priming the plants’ resistance to pathogen attack. Decreased accumulation of ROS in tomato upon pathogen attack. Enhanced expression of similar tomato genes as following *Botrytis* infection.	Finiti et al., 2014
	*A. thaliana*	Soil^6^	*B. cinerea*	2 d	Induction of resistance to pathogen attack. Hx-induced resistance is dependent on JA but independent of ABA, SA, ET and glutathion signaling or callose deposition.	Kravchuk et al., 2011
	*Citrus clementina* grafted onto *Carrizo citrange* ^13^	Soil^6^	*A. alternata*	2 - 3 d	Activation of mevalonic and linolenic acid pathways upon pathogen attack. Hx stays in the root and induces distal resistance. Enhanced emission of volatile metabolites.	Llorens et al., 2016
**GABA**	*A. thaliana*	Spray^9^	*B. cinerea*	Up to 4 d	Decreased accumulation of ROS upon *Botrytis* infection involving increased activities of catalase and guaiacol peroxidase.	Janse van Rensburg and van den Ende, 2020
**LOS**	*Lactuca sativa* var. Gisela	Spray^9^	*B. cinerea*	3 d	Improvement of the plants’ resistance to pathogen attack involving the accumulation of hydrogen peroxide and GABA. ET signaling is essential.	Tarkowski et al., 2019 and Tarkowski et al., 2020
	*A. thaliana*	Spray^9^	*B. cinerea*	3 d	Inulin and levan oligosaccharide (LOS) enhance plant defense upon pathogen exposure. Fructan pre-treatment enhances hydrogen peroxide accumulation and increases the activity of antioxidant enzymes (catalase, ascorbate peroxidase) upon pathogen exposure. In addition, glucose, sucrose and total soluble sugars accumulate in plants pre-treated with fructans.	Janse van Rensburg et al., 2020

^1^App, application of chemical priming agents; ^2^Lag, time lag between chemical treatment and pathogen infection; ^3^Medium, compounds were added into the media; ^4^Time period between compound addition into the media and the pathogen or the elicitor treatment. It is arguable whether this represents a lag phase; ^5^Duration entire plant pots were submerged into the SA solution. It is arguable whether this represents a lag phase; ^6^Soil, soil-drenching; ^7^Leaf inf, leaf infiltration; ^8^Rep, repotting of plants after chemical priming; ^9^Spray, spraying of chemical priming agent; ^10^Root, root-drenching. ^11^NL, no lag phase; ^12^Time leaves were fed with CK. It is arguable whether this represents a lag phase; ^13^ *Citrus clementina* grafted onto *Carrizo citrange*, *Citrus clementina* (hort. ex Tanaka x Dancy mandarin) grafted onto *Carrizo citrange* plants (*Citrus sinensis* L. Osbeck x *Poncirus trifoliata* Blanco).

Pep-13, elicitor-active 13-amino acid oligopeptide derived from the Pmg elicitor; TMV, tobacco mosaic virus; Pmg, cell wall elicitor from *Phytophthora megasperma* f. sp. *glycinea*; *Psg*, *Pseudomonas syringae* pv. *glycinea*; sc, suspension culture; *Pph, P. syringae* pv. *phaseolicola*; *Pss*, *P. syringae* pv. *syringae*; *Pst*, *P. syringae* pv. *tomato*; *Pstb*, *P. syringae* pv. *tabaci*; *Psm*, *P. syringae* pv. *maculicola*; *Hpa, Hyaloperonospora parasitica*; AZA, azelaic acid; BABA, beta-aminobutyric acid; BAP, 6-benzylaminopurine; BTH, benzothiadiazole; GABA, gamma- aminobutyric acid; Hx, hexanoic acid; INA, 2,6-dichloroisonicotinic acid; KIN, kinetin, 6-furfurylaminopurin; LOS, inulin and levan oligosaccharide; MeJA, methyl jasmonate; NHP, N-hydroxypipecolic acid; Pip, pipecolic acid; SA, salicylic acid; tZ, trans-zeatin.

### 3.2 *N*-hydroxypipecolic acid and pipecolic acid

**Figure 2:**
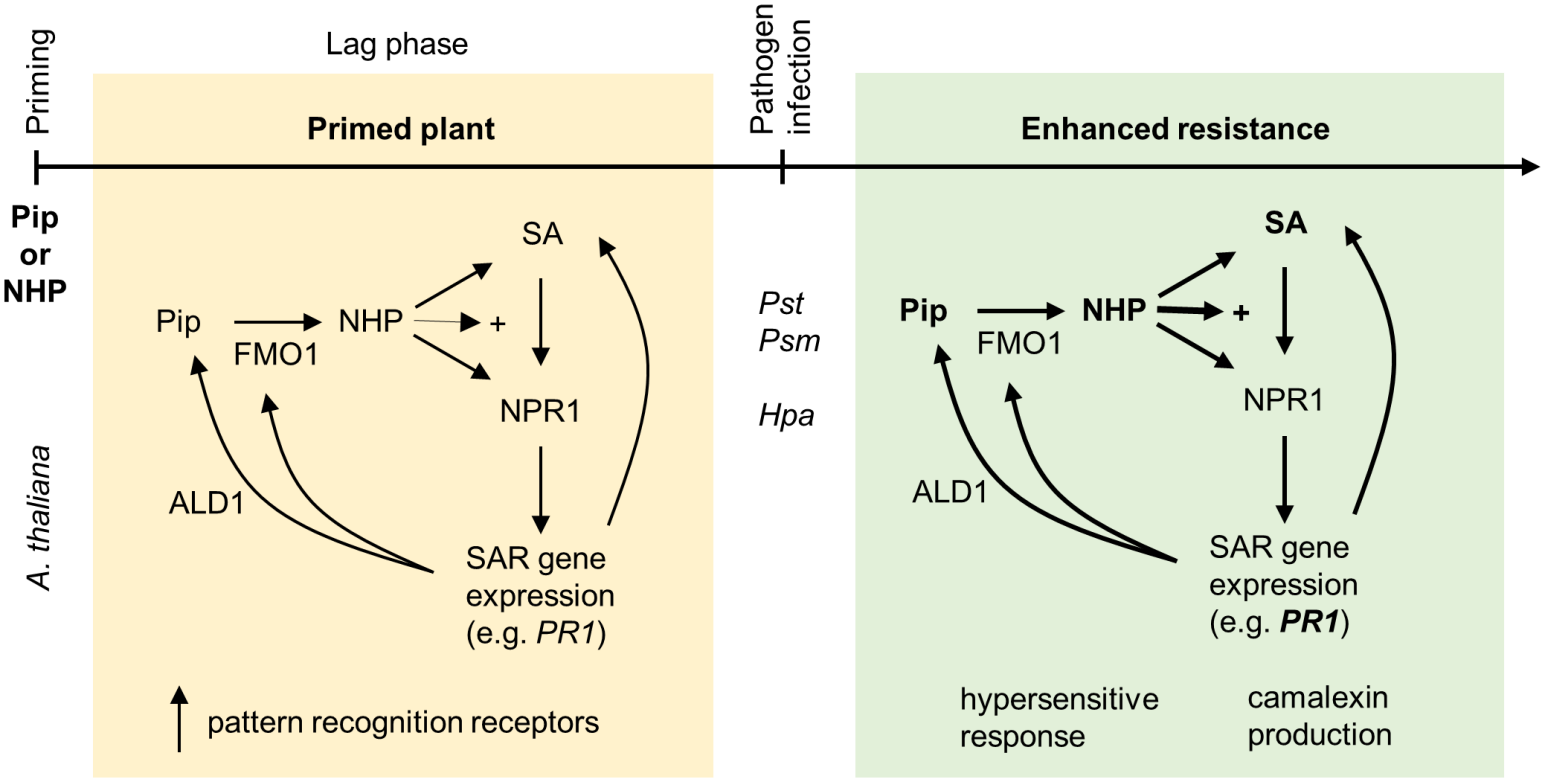
Priming by pipecolic acid and *N*-hydroxypipecolic acid. Application of NHP or its biological precursor Pip primes *A. thaliana* for enhanced pathogen resistance. Pip is metabolized in plants to NHP by the pipecolate *N*-hydroxylase FLAVIN-DEPENDENT MONOOXYGENASE1 (FMO1). NHP accumulates in primed tissue and further induces its own biosynthesis as well as the production of SA through the transcriptional coregulator NPR1. Furthermore, NHP-treated plants equip themselves with pattern recognition receptors. Induction of defense genes takes place directly after NHP application; however, the effect is low in comparison with the consequences of an infection with *Psm* and/or *Hpa*. The SA-driven response to pathogen infection, which is dependent on NPR1, is enhanced (indicated by +) due to the presence of NHP. In addition, NHP stimulates a SA-independent transcriptional response. Together, this primed response is manifested by elevated expression of pathogen-responsive genes, camalexin production and induction of a hypersensitive response. The figure has been modified from Yildiz et al. (2021).

NHP and Pip are important endogenous plant immune modulators that possess priming activity (**Table 1**, **Figure 2**) when exogenously applied to various plants (Guerra and Romeis, 2020). Pip pretreatment increased the resistance to bacteria, SA biosynthesis and camalexin production. Concurrently, higher pathogen-triggered expression of *AGD2-LIKE DEFENSE RESPONSE PROTEIN1* (*ALD1*, pipecolic acid biosynthetic pathway gene), *FLAVIN-DEPENDENT MONOOXYGENASE1* (*FMO1*) and *PR1* was observed in *Arabidopsis* (Návarová et al., 2012). Wang et al. (2018) showed that exogenous application of Pip confers SAR through induction of NO, ROS, AZA and G3P accumulation. However, whether the induced defense response is rather direct or primed was not studied. Priming by Pip was also detected in tobacco plants and was manifested as a rapid and strong accumulation of SA and nicotine following *P. syringae* pv. *tabaci* (*Pstb*) infection (Vogel-Adghough et al., 2013). Pip was found to orchestrate the SA-dependent and partially SA-independent SAR transcriptional response of defense genes. This response relied on FMO1, which functions as a pipecolate *N*-hydroxylase, catalyzing the biochemical conversion of Pip to NHP (Bernsdorff et al., 2016; Hartmann et al., 2018). Chen et al. (2018) showed that NHP but not Pip pretreatment of *Arabidopsis* lower leaves reduced the amount of *P. syringae* pv. *maculicola* (*Psm*) ES4326 and symptom development in infected upper leaves suggesting that NHP (or a NHP-induced transmitter) moves systemically in *Arabidopsis* to enhance resistance to this bacterial pathogen (Chen et al., 2018). NHP treatment enhanced pathogen-triggered activation of defense metabolism, including biosynthesis of SA, Pip, branched-chain amino acids and the phytoalexin camalexin, and increased the hypersensitive response. NHP also conditioned *Arabidopsis* for a stronger SA- and pathogen-induced expression of defense-related genes. It has been shown that the NHP priming effect required the function of the transcriptional coregulator NPR1 (Chen et al., 2018; Hartmann et al., 2018; Yildiz et al., 2021).

Both, Pip and NHP, were found to trigger IR, more specifically SAR, also in crop plants. Exogenous application of Pip induced systemic resistance in barley against the hemi-biotrophic pathogen *Xanthomonas translucent* pv. *cerealis*. Besides direct induction of NO after Pip treatment, priming for enhanced accumulation of superoxide anion was observed (Lenk et al., 2019). Application of NHP induced resistance against bacterial infection also in *Cucumis sativum* and *Nicotiana tabacum* as well as in the model plant for cereal crops *Brachypodium distachyon* (Schnake et al., 2020). Most recently, Pip biosynthesis was found to be crucial for SAR and plant-to-plant-induced immunity in barley (*Hordeum vulgare*). It was shown that in *Hvald1* biosynthetic mutants not only the systemic defense was altered, but also the emission of nonanal, one of the key volatile organic compounds (VOCs) that are normally emitted by barley plants after the activation of SAR (Brambilla et al., 2023).

Pip and especially NHP seem to be good candidates to function as alternatives for conventional fungicides and pesticides. NHP serves as a defense signaling molecule in various dicot and monocot plants (Schnake et al., 2020) and due to its priming activity, it has the potential to provide a resource-efficient resistance. However, various mutants with constitutively elevated concentrations of NHP possess not only an enhanced resistance but also a dwarf phenotype. When the ability to synthesize NHP was blocked in these mutants, the enhanced resistance and dwarfism were lost (Guerra et al., 2020; Mohnike et al., 2021). This indicated that maintaining the balance in NHP concentration is crucial for regulating growth-defense trade-offs (Bauer et al., 2021; Cai et al., 2021; Hõrak, 2021; Mohnike et al., 2021; Shields et al., 2022). In *Arabidopsis*, NHP homeostasis is modulated by glycosyltransferase UGT76B1 catalyzing the formation of NHP-OGlc and thus regulating and maintaining the balance between plant growth and defense response (Mohnike et al., 2021).

### 3.3 Azelaic acid

AZA, a lipid-derived saturated dicarboxylic acid, has been described as a mobile priming signal that is able to prime both local and systemic resistance to *P. syringae* (Jung et al., 2009) (**Table 1**). Plants pretreated with AZA for 12 to 48 hours and subsequently infected with *P. syringae* had higher levels of both SA and transcripts of the SA-associated signaling marker *PR1* compared with mock-treated plants (Jung et al., 2009). Notably, in another study an only marginal and not always reproducible potentiation of *PR1* expression was observed (Wittek et al., 2014). AZA requires the DEFECTIVE IN INDUCED RESISTANCE1 (DIR1) protein, the lipid transfer protein (LTP)-like AZELAIC ACID INDUCED1 (AZI1) and its closest paralog EARLY *ARABIDOPSIS* ALUMINIUM INDUCED1 (EARLI1) to prime plants for SAR (Jung et al., 2009; Yu et al., 2013; Cecchini et al., 2015). Pools of AZI1 and EARLI1 are localized in the plastid envelopes, primarily in epidermal cells (Banday et al., 2022) and support the mobilization of plastid-produced AZA (Zoeller et al., 2012; Cecchini et al., 2015). Furthermore, accumulation of AZI1 in plastids is promoted by MPK3/6 (Cecchini et al., 2021), important regulators of primed stress responses. The priming effect of AZA was impaired in various SA synthesis and signalling mutants (*pad4*, *ndr1*, *npr1*, *sid1*, *sid2*, *dth9*) and Pip/NHP mutants (*ald1*, *fmo1*) but was not impaired in glycerolipid mutants (*fad7*, *sfd1*) lacking glycerolipid-requiring SAR signals (Jung et al., 2009; Cecchini et al., 2015), indicating that AZA-priming also requires SA biosynthesis and signaling. AZA appeared to be a mobile compound when applied *via* leaves. Uptake and movement of labeled AZA from one leaf (the application site) to other tissues (aerial stem/leaves and roots) was significantly reduced in *azi1-1* and *earli1-1* compared to WT plants, but not in *dir1-1* (Cecchini et al., 2015), indicating that AZI1 and EARLI1 are required for the transport. In contrast, root-applied AZA did not move upward to aerial tissues and did not prime for the induction of PR1 and LOX2 proteins after bacterial infiltration (Cecchini et al., 2019).

### 3.4 Methyl jasmonate

**Figure 3:**
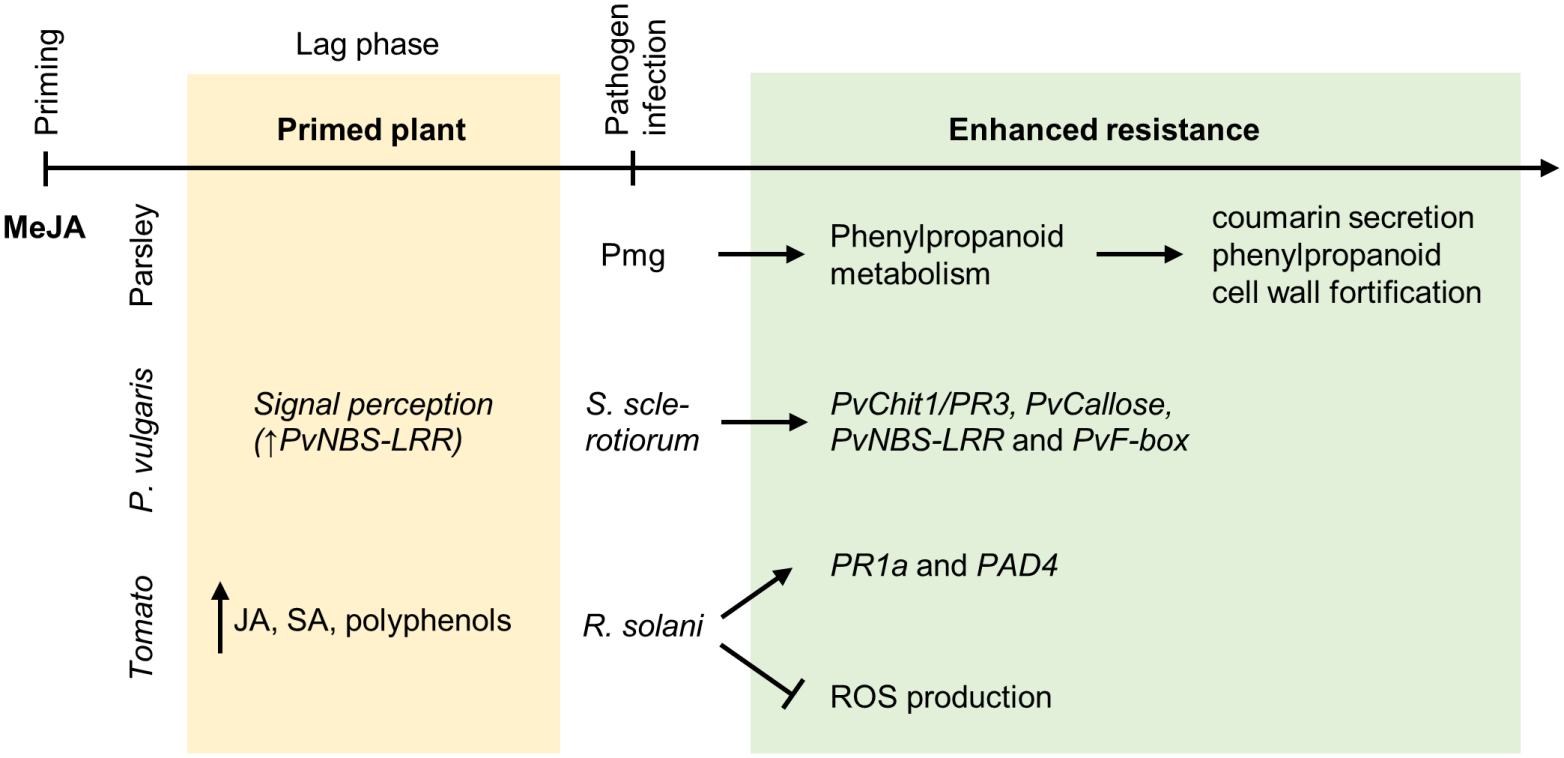
Priming by methyl jasmonate. MeJA is the most efficient JA compound for chemical priming. Primed *P. crispum* L. (parsley) suspension culture cells show upon triggering by the elicitor Pmg an augmented secretion of coumarin derivatives as well as cell wall fortification by incorporation of hydroxycinnamic acids and “lignin-like” polymers. *NBS-LRR* resistance-like protein gene expression is significantly increased in *Phaseolus vulgaris* L. plants treated with MeJA. This suggests an enhanced signal perception in plants before their infection with *Sclerotinia sclerotiorum* (Lib.) de Bary. The infection is followed by an elevated expression of the pathogen-related gene *PvChit1/PR3*, the JA signaling gene *PvF-box* and the callose deposition-related gene *PvCallose*. Primed tomato plants show an increased biosynthesis of both SA and JA, together with polyphenols. After the infection with the necrotrophic pathogen *R. solani*, augmented expression of *PR1a*, and of the *PAD4* gene was observed. MeJA pretreatment further limits the ROS production stimulated by *R. solani* infection.

In addition to JA, its volatile derivative MeJA plays also vital roles in plant responses to biotic and abiotic stresses. Direct application of MeJA was shown to improve the resistance of plants to biotic stresses (**Table 1**, **Figure 3**). MeJA pretreatment of suspension-cultured parsley cells enhanced their ability to respond to a fungal elicitor (Pmg) by secretion of coumarin derivatives and incorporation of esterified hydroxycinnamic acids and “lignin-like” polymers into the cell wall. This indicated a general effect on phenylpropanoid metabolism (Kauss et al., 1992a). MeJA pretreatment of dry bean plants (*Phaseolus vulgaris* L.) significantly delayed symptoms of infection by the necrotrophic fungus *Sclerotinia sclerotiorum* (Lib.) de Bary (Oliveira et al., 2015). In addition, Pieterse et al. (1998) reported that MeJA pretreatment could be used for priming in *Arabidopsis* and revealed the importance of NPR1 in this process. In dry bean plants, MeJA pretreatment was shown to cause a systemically upregulated expression of several defense-related genes, such as *PvPR3-CHITINASE CLASS1* (*PvChit1*), the callose synthase gene *PvCallose*, the NBS-LRR resistance-like protein gene *PvNBS-LRR* and the gene F-box family protein-like *PvF-box*, after subsequent infection with the fungus *S. sclerotiorum*. MeJA treatment alone only induced the expression of *PvNBS-LRR* (Oliveira et al., 2015). The authors concluded that MeJA pretreatment enabled transcriptional reprogramming during their early response to *S. sclerotiorum* and thereby enhanced plant defense responses (Oliveira et al., 2015). Recently, the necrotrophic pathogen *Rhizoctonia solani* has been reported to be able to distinguish MeJA- and SA-primed tomato plants from a distance and avoided SA-primed plants more than MeJA-primed plants (Koley et al., 2022). Overall, in these experiments MeJA provided a slower and weaker protection compared with SA (Koley et al., 2022).

With respect to horticultural use, a priming mechanism was suggested for the protective effect of MeJA in Chinese bayberries (*Myrica rubra* Seib and Zucc. Cv. Wumei) (Wang et al., 2014) against *Penicillium citrinum*, resulting in improved postharvest fruit preservation.

### 3.5 Beta-aminobutyric acid

**Figure 4:**
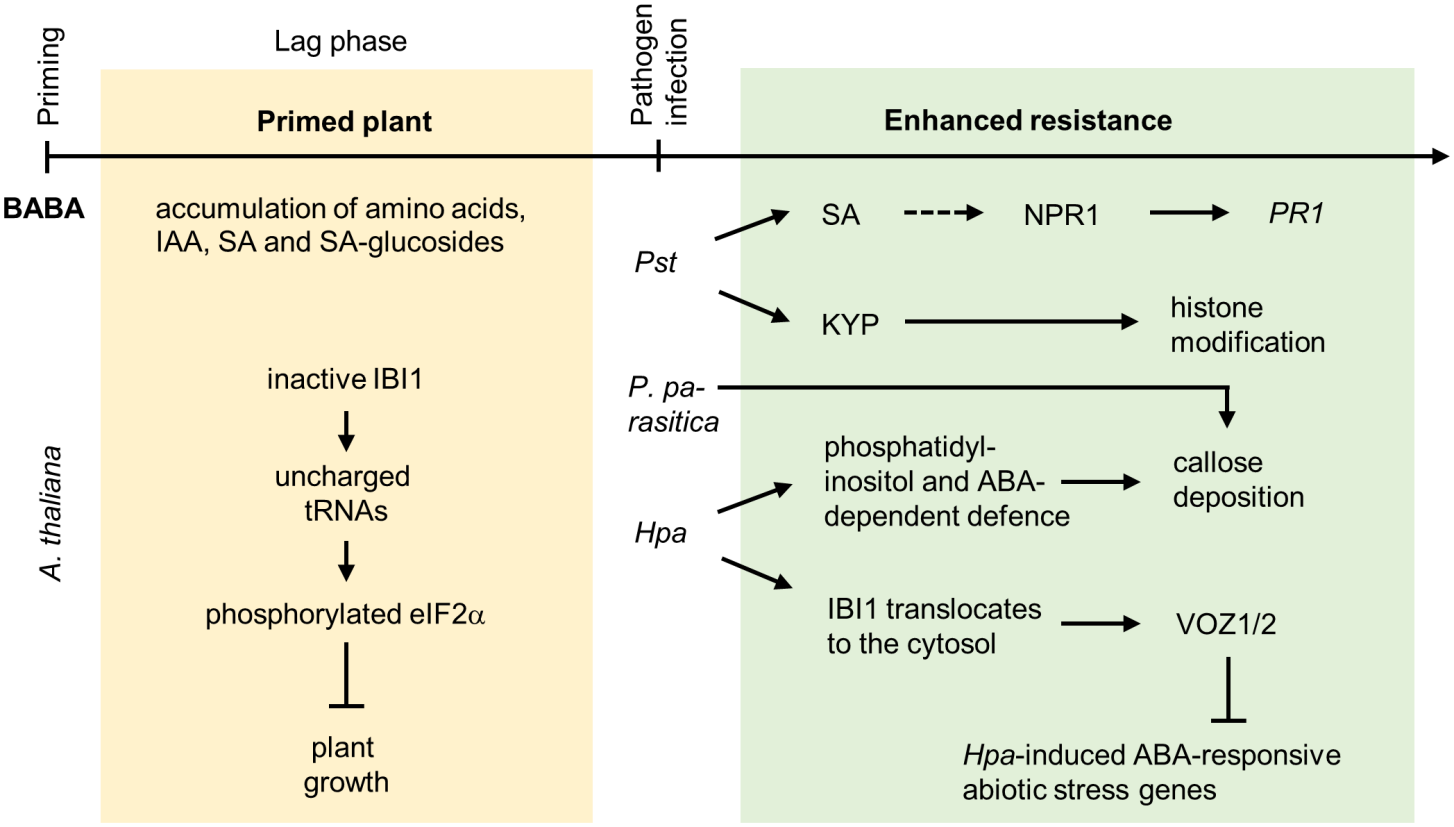
Priming by β-aminobutyric acid. In *Arabidopsis*, the *R* enantiomer of BABA, which is structurally similar to *L*-aspartic acid, is perceived by IMPAIRED IN BABA-INDUCED IMMUNITY1 (IBI1) representing an aspartyl-transfer RNA (tRNA) synthetase located in the endoplasmic reticulum. Binding of BABA inhibits the activity of IBI1. This increases the content of uncharged tRNAs, which causes the phosphorylation of the translation initiation factor eukaryotic Initiation Factor 2 subunit alpha (eIF2α). This leads to BABA-mediated suppression of plant growth and reproduction. Besides this inhibitory effect on plant growth, BABA primes the production of amino acids, IAA, SA and SA-glucosides, and xanthosine. BABA enhances the plant primary metabolism by induction of compounds belonging to the tricarboxylic acid cycle, potentiates the phenylpropanoid and phenylpropanoid biosynthesis and the octodecanoic pathway. Depending on the pathogen attacking *Arabidopsis*, priming by BABA involves SA- and SAR-dependent or ABA-dependent signaling pathways. In the case of *Pst* infection, BABA primed resistance involving a potentiated expression of the defense gene *PATHOGENESIS-RELATED PROTEIN1* (*PR1*), which is regulated through NONEXPRESSOR OF PATHOGENESIS-RELATED GENES1 (NPR1). NPR1 is especially important for long-term priming by BABA (dashed line arrow), short-term priming is independent of NPR1. The primed state is maintained by the histone methyltransferase SUVH4/KRYPTONITE (KYP), pointing to the involvement of epigenetic regulations. Interestingly, neither SA nor SAR are important for priming by BABA to infections with the oomycetes *P. parasitica* or *Hpa*. Priming by BABA against *Hpa* infection involves phosphatidylinositol- and ABA-dependent defense responses resulting in increased callose deposition. In addition, BABA primes the *Hpa*-induced translocation of IBI1 from the endoplasmic reticulum to the cytosol, which results in the interaction of IBI1 with defense regulators, such as the transcription factors VASCULAR PLANT ONE ZINC FINGER1 (VOZ1) and VOZ2. VOZ1/2 suppress the *Hpa*-induced expression of ABA-responsive abiotic stress genes and enhances VOZ1/2-dependent expression of genes regulating (early) PTI.

One of the most prominent chemical inducers of plant defense responses to various biotic stressors is β-aminobutyric acid (BABA) (Conrath et al., 2006; Walters et al., 2013; Schwarzenbacher et al., 2014; Cohen et al., 2016). Already in 1963, Papavizas and Davey described that BABA induces resistance to the oomycete *Aphanomyces euteiches* (Papavizas and Davey, 1963). The following work showed that BABA-induced resistance (BABA-IR) protects diverse plant species from an immense range of pathogens (Cohen et al., 2016). BABA-IR to biotic stresses involves priming of SA-dependent but also -independent defense responses (Cooper and Ton, 2022) (**Figure 4**).

BABA is a non-proteinogenic amino acid that had long been considered a xenobiotic in plants in contrast to its isomers α-aminobutyric acid (AABA) and γ-aminobutyric acid (GABA). However, Thevenet et al. (2017) recently showed that BABA is naturally present in plants and accumulates in response to both abiotic and biotic stresses, which is precisely controlled by the plants’ defense system (Baccelli et al., 2017; Balmer et al., 2019). Resistance induced by BABA is highly stereospecific. Only the *R* enantiomer of BABA is active in plants and enhances the plant defense against biotic stress, while the *S* enantiomer of BABA, as well as the isomer AABA, do not induce the plants’ resistance (Siegrist et al., 2000; Silué et al., 2002; Thevenet et al., 2017).

In *Arabidopsis*, BABA is perceived by IMPAIRED IN BABA-INDUCED IMMUNITY1 (IBI1) representing an aspartyl-tRNA synthetase located in the endoplasmic reticulum. IBI1 binds the *R* enantiomer of BABA (due to its *L*-aspartic acid resembling structure), which inhibits IBI1 enzyme activity. The inhibition of IBI1 by BABA increases the content of uncharged tRNAs, which causes the phosphorylation of the translation initiation factor eIF2α (Luna et al., 2014b; Cooper and Ton, 2022). Thereby BABA treatment suppresses plant growth and reproduction, which points to a BABA-related trade-off between inducing plant defense while inhibiting growth. Nevertheless, BABA is a promising chemical priming agent as it improves plant defense responses to various pathogens (**Table 1**).

In *Arabidopsis*, priming by BABA efficiently enhanced resistance to *Pst* DC3000 as well as to the oomycetes *Hyaloperonospora parasitica* (*Hpa*) and *Peronospora parasitica* (**Table 1**). Common to different studies with *Arabidopsis* in which BABA primes resistance to *Pseudomonas* bacteria is the induction of SA signaling, including a potentiated *PR1* induction upon pathogen exposure (Zimmerli et al., 2000; Ton et al., 2005; van Hulten et al., 2006; Wu et al., 2010; Slaughter et al., 2012). BABA treatment itself is not sufficient to induce *PR1* gene expression (Zimmerli et al., 2000). Interestingly, the concentration of the applied BABA determines the outcome of the primed defence response: Low concentrations of BABA prime systemic defence responses, while higher concentrations rather directly activate defence responses in the treated tissues (van Hulten et al., 2006; De Kesel et al., 2021). Especially long-term priming by BABA involved SA-regulated defense genes and NPR1, while short-term BABA-induced resistance is independent of NPR1 (Luna et al., 2014a). Comparisons of BABA- and *Pseudomonas*-treated *Arabidopsis* revealed that BABA treatment primed the production of amino acids and phytohormones, including indole acetic acid, SA and SA glucosides (Pastor et al., 2014). Mutant analysis confirmed that priming by BABA against *Pseudomonas* infection required SA and SAR signaling. In contrast, neither SA nor SAR signaling is essential for BABA-induced priming against infection with *Peronospora parasitica*, indicating that BABA induces different responses to protect against different pathogens (Zimmerli et al., 2000). Pathogen-specificity of the mechanisms involved in priming by BABA is further supported by observations of Ton et al. (2005), who described that priming against *Hpa* involved phosphatidylinositol- and ABA-dependent defense responses resulting in increased callose deposition, which was not reported for *Pst*-infected *Arabidopsis* plants. Schwarzenbacher et al. (2020) showed that BABA primed the *Hpa*-induced translocation of IBI1 from the endoplasmic reticulum to the cytosol, leading to the interaction of IBI1 with defense regulators, such as VASCULAR PLANT ONE ZINC FINGER1 (VOZ1) and VOZ2 transcription factors. Activated VOZ1/2 suppresses *Hpa*-induced expression of ABA-responsive abiotic stress genes and enhances VOZ1/2-dependent expression of genes regulating (early) PTI (Schwarzenbacher et al., 2020; Cooper and Ton, 2022). Neither JA nor ET signaling is required for the priming of *Arabidopsis* by BABA against *Pseudomonas* or oomycete infection (Zimmerli et al., 2000).

BABA also primes the pathogen defense of other plants than *Arabidopsis*, including potato (*Solanum tuberosum*) (Bengtsson et al., 2014), tomato (*Solanum lycopersicum*) (Luna et al., 2016; Catoni et al., 2022), common bean (*Phaseolus vulgaris*) (Martínez-Aguilar et al., 2016), tobacco (*Nicotiana tabacum*) (Ren et al., 2022), grapevine (*Vitis vinifera*) (Hamiduzzaman et al., 2005) or lettuce (*Lactuca sativa*) (Chalupowicz et al., 2021). Similarly as described for *Arabidopsis*, BABA treatment potentiated *PR1* expression in *L. sativa* exposed to *Salmonella enterica serovar Typhimurium* (Chalupowicz et al., 2021), in *V. vinifera* infected with *Plasmopara viticola* (Hamiduzzaman et al., 2005) or in *P. vulgaris* infected with *P. syringae* pv. *phaseolicola*. In common bean, in addition, enhanced induction of *PR4*, *NPR1*, *WRKY6*, *WRKY29* and *WRKY53* after pathogen infection was observed in BABA-pretreated plants, while BABA treatment alone did not result in induction of these genes indicating priming (Martínez-Aguilar et al., 2016). As described for *Arabidopsis*, BABA influences processes connected to phytohormones and amino acid metabolism in tomato plants (Bengtsson et al., 2014). This suggests that BABA priming involves similar signaling pathways in different plant species. However, this was not the case in all priming experiments performed with BABA. For instance, BABA-priming of tomato plants prior to infection with *Hpa* did not involve ABA-responsive genes (Bengtsson et al., 2014), although an ABA-dependency has been described for *Arabidopsis* (Ton et al., 2005). In addition to the improved resistance to *Pseudomonas* bacteria or oomycetes in different plant species, BABA primes also SA- and JA-dependent resistance against the necrotrophic fungus *Botrytis cinerea* in tomato (Luna et al., 2016; Catoni et al., 2022).

Recent research indicates that priming by BABA involves epigenetic regulation as well (Cooper and Ton, 2022). In *Arabidopsis*, the histone methyltransferase SUVH4/KRYPTONITE (KYP) is required for maintaining the primed state. Regarding histone modifications, no long-term effects on H3K9ac levels at the promoters of *PR1* or *WRKYs* were observed (Luna et al., 2014a). In common bean, BABA increased H3K4me3 and H3K36me3 chromatin marks on the promoter of *PR1* and H3K4me3 chromatin marks at the promoter-exon boundary region of *WRKY6* and *WRKY29* 24 hours after BABA treatment, although no transcriptional induction of the respective genes was observed. Both chromatin marks decreased upon infection with *P. syringae* pv. *phaseolicola* in BABA-primed plants, while gene expression was induced. This indicates a biphasic behavior typical for priming (Martínez-Aguilar et al., 2016). In contrast to these specific changes, BABA priming resulted in tomato in genome-wide DNA methylation (Catoni et al., 2022).

Although chemical priming by BABA has been described for several plant species and efficiently induces plant defense responses to different biotic stressors, BABA is not commonly used in agriculture due to its negative effects on plant growth (Wu et al., 2010; Cooper and Ton, 2022). In *Arabidopsis*, these negative effects were decreased by treatment with *L*-glutamine. However, *L*-glutamine also removed the primed resistance induced by BABA (Wu et al., 2010). The identification of IBI1 as a BABA-receptor and increasing understanding of the mechanisms involved in priming by BABA offer new possibilities for crop breeding and genetically design plants with a primed immune state (Cooper and Ton, 2022). In peach fruits, priming by BABA was recently suggested as a postharvest strategy to improve the resistance to the necrotrophic fungus *Rhizopus stolonifera* causing soft rot on peach fruit surfaces (Li et al., 2021). Priming by BABA enhanced the hydrogen peroxide content in postharvest-infected peach fruits and boosted the mitogen-activated protein kinase (MAPK) cascade involving PpMAPKK5, which interacts with the transcription factor PpTGA1, thereby activating SA-dependent responses and decreasing the fungus-induced disease symptoms (Li et al., 2021). A similar postharvest improvement of the resistance to *Penicillium italicum* was observed in fruits of sweet orange *Citrus sinensis* (Tavallali et al., 2008), underpinning the possibility of using BABA treatment in harvested fruits. The investigation of direct effects of BABA on pathogens might be of interest for future research as well. Ren et al. (2022) recently showed that BABA itself directly restricts the growth of the oomycete *Phytophthora parasitica*.

### 3.6 Gamma-aminobutyric acid

The non-proteinogenic amino acid γ-aminobutyric acid (GABA) regulates responses to abiotic and biotic stresses (Janse van Rensburg and van den Ende, 2020; Tarkowski et al., 2020). Although earlier studies indicated that GABA does not induce resistance to pathogens in tobacco (Siegrist et al., 2000) or cauliflower (Silué et al., 2002), recent research suggests that GABA primes the resistance to *B. cinerea* in *A. thaliana* (Janse van Rensburg and van den Ende, 2020). Interestingly, priming by GABA decreases the accumulation of ROS upon *Botrytis* infection, involving increased activities of catalase and guaiacol peroxidase (**Table 1**). This indicates that GABA priming utilizes the plant redox system. In addition, GABA treatment promotes the metabolic activity of *Arabidopsis* during *Botrytis* infection by inducing the accumulation of soluble sugars (Janse van Rensburg and van den Ende, 2020). However, research suggesting priming by GABA in plants is rather limited, offering possibilities for future investigations.

### 3.7 Cytokinins

Cytokinins (CKs) are phytohormones regulating various aspects of plant growth and development, such as cell division, meristem activity and leaf senescence (Schaller et al., 2014), as well as responses to biotic and abiotic stresses (Cortleven et al., 2019), and function as chemical priming agents (**Table 1**). In *Arabidopsis*, CK modulates SA signaling to augment resistance against *Pst*. The CK-activated transcription factor ARR2 was shown to bind to TGA3 and activate the expression of *PR1* and the SA biosynthesis gene isochorismate synthase 1 (*ICS1).* The binding of ARR2 and TGA3 to the *PR1* promoter was dependent on NPR1 (Choi et al., 2010). In the same study, *trans*-zeatin (tZ) pretreatment for 24 h was shown to potentiate the activation of *PR1* gene expression upon bacteria inoculation, in addition to the direct activation of defense-related genes through ARR2 (Choi et al., 2010). In another study, pretreatment with 6-benzylaminopurin (BAP) enhanced the resistance of *Arabidopsis* against the virulent oomycete *Hpa* isolate Noco2 in a dose‐dependent manner. While SA-responsive defense genes were only slightly up-regulated in response to BA pretreatment, *Hpa* Noco2 inoculation led to a further enhancement of the expression of these genes. This induction was decreased in the SA biosynthesis mutants *eds16* (Argueso et al., 2012). Notably, in both studies, CK pretreatment did not augment pathogen resistance in the SA signaling mutant *npr1* nor in the SA deficient mutant *eds16* (Choi et al., 2010; Argueso et al., 2012) corroborating the relevance of the SA pathway for priming by CK. Feeding tobacco leaves with various CKs prior to bacterial infection strongly enhanced the resistance to the hemibiotrophic pathogen *Pstb* (Großkinsky et al., 2011). In this study, the CK effect was associated with a high phytoalexin-pathogen ratio in the early phase of infection rather than to an effect on SA signaling, suggesting a different mechanism from *Arabidopsis* (Großkinsky et al., 2011).

More recently it was reported that foliar application of certain CK sugar conjugates, CK-arabinosides (CK-A), to field-grown wheat and barley plants decreased infection by several fungal pathogens. Furthermore, RNAseq and gene expression studies showed that CK-As might operate through inducing a PTI response in *A. thaliana*. It was suggested that the enhanced defensive capacity against fungal pathogens is due to priming of IR (Bryksová et al., 2020).

### 3.8 Hexanoic acid

**Figure 5:**
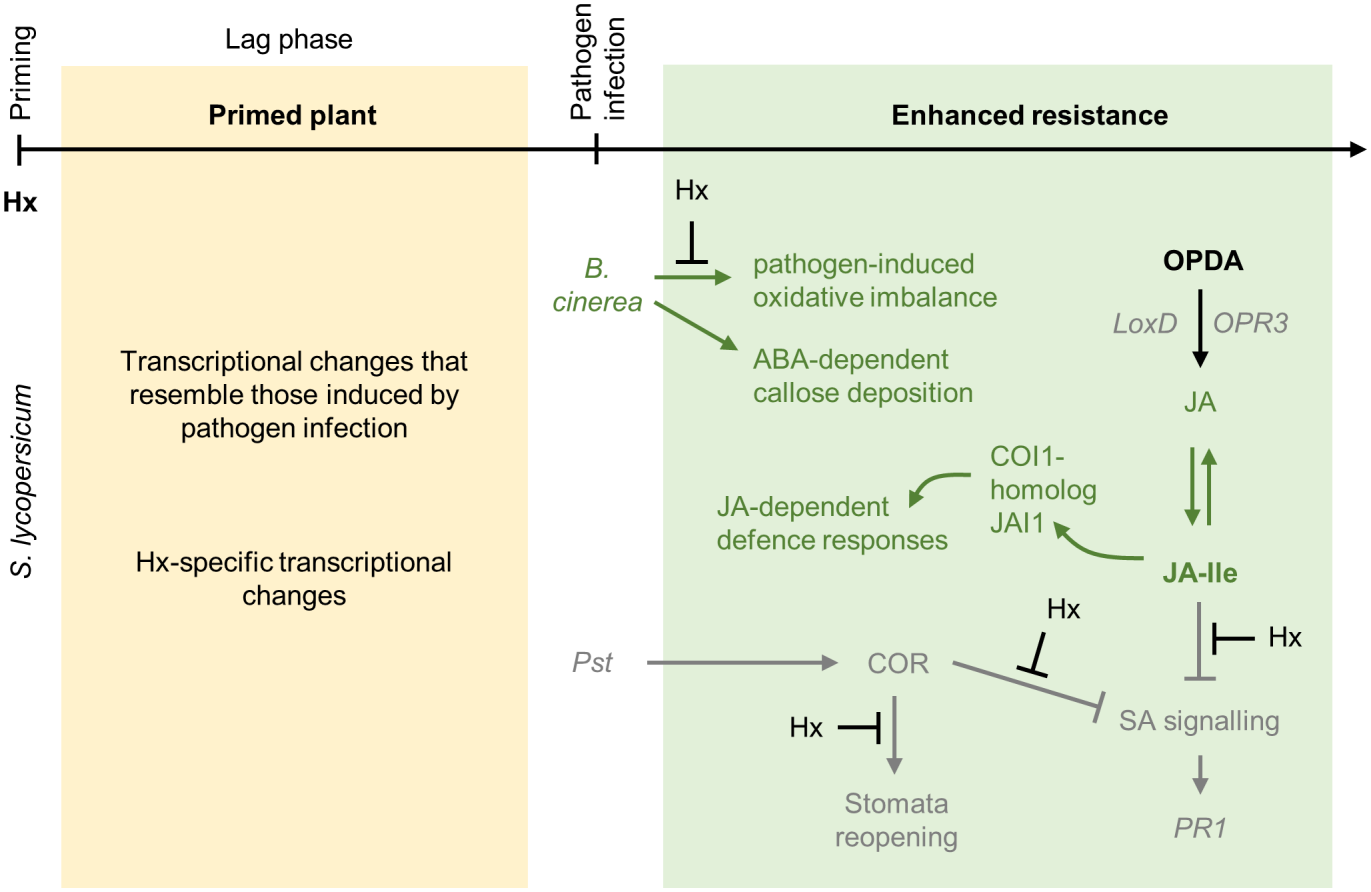
Priming by hexanoic acid. Hx is a monocarboxylic acid that functions as a natural priming agent in different plant species. The mechanisms enabling priming by Hx are best described in tomato plants. Exogenously applied Hx does not accumulate in tomato plants but still positively influences the plants’ resistance to pathogen infections. In tomato, Hx induces the expression of genes that are typically regulated in response to *Botrytis cinerea* infection (in addition genes are induced that respond specifically to Hx). Hx counteracts the pathogen-induced imbalance of the plant redox state, thereby enhancing the plants’ resistance. Especially JA signaling is potentiated by Hx treatment upon pathogen exposure. In response to *B. cinerea* infection (dark green color), Hx-primed tomato plants accumulate JA-isoleucine (JA-Ile) and 12-oxo-phytodienoic acid (OPDA) and increase the expression of the JA marker gene *LoxD*, while the expression of marker genes for SA and ET signaling is not enhanced. In addition, priming by Hx against *B. cinerea* requires the COI1-homolog JAI1 to induce downstream responses. Furthermore, in Hx-primed plants ABA signaling enhances callose deposition. This is not observed in Hx-primed tomatoes infected with *Pst* (grey color), indicating that priming by Hx activates different mechanisms depending on the pathogen. This is further supported by the observation that in response to *Pseudomonas* infection, priming by Hx requires functional SA signaling. Hx-priming increases the expression of SA signaling genes, such as *PR1* and *PR5*, upon pathogen infection. Hx-priming inhibits stomatal reopening, which is mediated by the bacterial-derived coronatine (COR) mimicking plant JA-Ile. Thereby, Hx likely prevents the bacteria from entering the plant mesophyll. In addition, Hx counteracts negative effects on SA signaling, which are mediated by COR and JA-Ile, and thus improves the plants’ resistance. Similar as *Botrytis*-infected tomato plants, Hx-primed tomato plants infected with *Pst* accumulate OPDA (but not JA or JA-Ile) and induce the expression of OPDA- and JA biosynthesis and signaling genes, such as *LoxD*, *OPR3*, and *JAZ1*.

Hexanoic acid (Hx) is an endogenous monocarboxylic acid that is structurally similar to green leafy volatiles (GLVs) (Aranega-Bou et al., 2014; Brilli et al., 2019). Hx is known to function as a natural priming compound (**Table 1**, **Figure 5**). More specifically, in tomato and *Arabidopsis*, Hx improved the resistance to *Botrytis cinerea* (Vicedo et al., 2009; Kravchuk et al., 2011; Finiti et al., 2014). In tomato, Hx treatment caused a decreased reactive oxygen species (ROS) accumulation (Finiti et al., 2014) and decreased necrosis development (Leyva et al., 2008) upon *Botrytis* infection. Additionally, Hx regulated and further potentiated the expression of similar genes as *Botrytis* infection, such as *PR1* (Finiti et al., 2014). Analysis of different tomato cultivars showed that Hx treatment potentiates the expression of the JA marker gene *lipoxygenase D* (*LoxD*) upon *Botrytis* infection, while expression of SA and ET signaling marker genes reached similar levels as control-treated plants. Experiments showed that Hx treatment primes faster and stronger accumulation of JA-isoleucine (JA-Ile) and 12-oxo-phytodienoic acid (OPDA), which are both known to mediate plant defense against fungal infection (Vicedo et al., 2009). Hx did not induce resistance in the JA-insensitive tomato mutant *jasmonic acid insensitive1* (*jai1*), strongly supporting the essential role of JA signaling for priming by Hx (Vicedo et al., 2009).

Hx treatment of tomato plants also improved the resistance to *P. syringae* infection (Vicedo et al., 2009; Scalschi et al., 2013). In that case, Hx prevented the bacteria from entering the plant mesophyll and concurrently counteracted negative effects on SA signaling and thus improved the plants’ resistance (Scalschi et al., 2013). Similar as described for Hx-treated tomatoes infected with *Botrytis*, expression of genes involved in OPDA- and JA biosynthesis and signaling, such as *LoxD*, *OPR3*, and *JAZ1*, was augmented (Scalschi et al., 2013). Hx also potentiated the expression of SA signaling genes, such as *PR1* and *PR5*, upon pathogen infection. The importance of a functional SA signaling pathway for Hx-mediated priming to protect against *Pseudomonas* was confirmed in experiments with transgenic *NahG* plants that are unable to accumulate SA and were not primed by Hx (Scalschi et al., 2013).

Besides its effect on JA and SA signaling, Hx also involves ABA signaling in tomatoes. The ABA-deficient tomato mutant *flacca* was not primed by Hx treatment for *Botrytis* infection (Vicedo et al., 2009). In addition, Hx treatment enhanced ABA-dependent callose deposition in tomato, suggesting that callose deposition may contribute to priming by Hx (Vicedo et al., 2009). Hx-induced accumulation of callose was also observed in *Arabidopsis* plants; however, this was not relevant to the priming process (Kravchuk et al., 2011).

Hx also induced resistance of Fortune mandarin plants grafted onto Carrizo citrange plants against *Alternaria alternata* infection (Llorens et al., 2016). Experiments with Hx labeled with ^13^C at the carboxylic end showed that Hx applied *via* soil-drenching stays in the roots and primes the plants for ISR. Hx treatment potentiated accumulation of defensive metabolites and volatile compounds emission (Llorens et al., 2016).

Reports on the practical use of Hx are to our knowledge lacking. However, because Hx treatment improves resistance to different plant pathogens, it seems worthwhile to explore its potential for future applications.

## 4 Artificial and other chemical priming compounds

In the following sections, we give a summary on artificial chemical priming compounds. We considered BTH (section 4.1), INA (section 4.2) and other chemical priming compounds (section 4.3).

### 4.1 Benzothiadiazole

BTH is a structural analog of SA with priming properties for improved SAR. Similar as SA, it was also first studied in parsley cell cultures (**Table 1**, **Figure 1**). BTH application potentiates *PAL* gene activation and enhances coumarin secretion after Pmg elicitor application. The augmentation of *PAL* gene induction was proportional to the duration of BTH pretreatment (Katz et al., 1998). Covalent modifications of histone H3 and chromatin opening in the *WRKY6* and *PR1* regulatory regions were found to be caused by BTH activity, together with an enhanced *WRKY6* induction upon flg22 treatment (Schillheim et al., 2018). *PAL* gene activation was also observed in BTH-treated *Arabidopsis*, along with enhanced callose production. NPR1 is necessary for BTH priming against *P. syringae* pv. *tomato* (*Pst*) DC3000 (Kohler et al., 2002), as well as MPK3/6 (Beckers et al., 2009). In rice (*Oryza sativa* cv. NB), BTH application potentiates diterpenoid phytoalexin biosynthesis upon *Magnaporthe oryzae* infection. The priming is regulated *via* SA/cytokinin synergism in a WRKY45-dependent manner (Akagi et al., 2014). An important role of *PAL* gene activation upon priming by BTH was further observed in the crop plant cowpea (*Vigna unguiculata*) (Latunde-Dada and Lucas, 2001). Seed treatment by BTH primed 7-days-old seedlings for enhanced resistance against *Colletotrichum destructivum.* The enhanced resistance was associated with rapid, transient increases in the activities of PAL and chalcone isomerase CHI, key enzymes of the phenylpropanoid/flavonoid pathway. Moreover, early, accelerated accumulation of the isoflavonoid phytoalexins kievitone and phaseollidin was observed after pathogen inoculation. These responses following inoculation were not observed in BTH-treated uninoculated tissues (Latunde-Dada and Lucas, 2001).

In the field, BTH application provided protection against a broad spectrum of diseases in a variety of crops and became an attractive compound for practical agronomic use (Ryals et al., 1996). However, BTH efficiency depends on several variables, such as the dose and frequency of application, host genotype (Vallad and Goodman, 2004), and, in one case, the plants’ growth stage (Heil et al., 2000). Together with the necessity to apply the agents in a preventive rather than curative manner, the economic success of BTH is limited, as farmers favor using standard curative fungicides (Conrath, 2009).

### 4.2 2,6-dichloroisonicotinic acid

In contrast to BTH, another SA structural analogue, namely 2,6-dichloroisonicotinic acid (INA), does not bind NPR1. INA was also shown to be a poor inducer of *PR1* expression in *Arabidopsis*. As there is still *PR1* expression after INA application, INA might activate *PR1* through a mechanism different from SA (Wu et al., 2012). Despite that, INA was described as a chemical priming agent in various experiments (**Table 1**). Pretreatment of parsley cell culture with INA augmented secretion of the phytoalexin coumarin (Kauss et al., 1992b). Similarly to SA, INA treatment of cucumber (*Cucumis sativus* L.) hypocotyls augmented hydrogen peroxide production upon treatment with elicitor from *Phytophora sojae* (Fauth et al., 1996). In *Phaseolus vulagris* L. INA showed the ability to potentiate the induction of *WRKY29* and *WRKY53* gene expression upon pathogen exposure (Martínez-Aguilar et al., 2016). Recently, it was shown that INA pretreatment primes common bean plants for increased resistance to *P. syringae* pv*. phaseolicola* (*Pph*) through cell wall remodeling increasing the plants’ resistance to enzymatic hydrolysis. In parallel, INA pretreatment produced the highest ROS peak after the addition of flg22 (which is argued to mimic *Pph* inoculation), showing that INA does not directly increase ROS production but primes the bean cells for stronger defense responses (De la Rubia et al., 2021).

### 4.3 Other chemical priming compounds

Although not addressed in detail here (as we concentrated on the more prominent examples for chemical priming of plant responses to pathogen attacks), also other compounds have the potential to prime plants against pathogen infections. Examples of these are biostimulants, such as seaweed-based biostimulants that are used since years to improve the performance of plants in agriculture (Nanda et al., 2022). For instance, the water-soluble menadione sodium bisulphite belonging to the vitamin K class of compounds primes defense responses to *Pst* in *Arabidopsis* plants (Borges et al., 2009; Borges et al., 2014). Additionally, *Zea mays* pretreatment by indole induced earlier and stronger expression of *PR* protein genes, JA (*LOX1*) and phytoalexin (*An2*) biosynthetic genes and antioxidant enzymes-encoding genes (*CAT1*, *POD1*) upon *F. graminearum* spores’ inoculation. This induction was dependent on the MAPK cascade and involved a ROS burst at the pretreatment stage (Shen et al., 2018).

Also fructans, which include oligo- and polysaccharides that are mainly composed of fructose rings, have the potential to prime plants. In land plants that are able to accumulate fructans, these are involved in responses to abiotic and biotic stresses (Tarkowski et al., 2019; Janse van Rensburg et al., 2020). Exogenous treatment of *Arabidopsis* plants with fructans like inulin and levan oligosaccharide (LOS) enhances their accumulation of hydrogen peroxide and increases the activities of the antioxidant enzymes ascorbate peroxidase and catalase following an infection with *B. cinerea* (Janse van Rensburg et al., 2020). Studies with leafy vegetable lettuce (*L. sativa*) suggested that treatment with the fructan inulin further increases the accumulation of hydrogen peroxide and GABA when infected with *B. cinerea* (Tarkowski et al., 2019; Tarkowski et al., 2020). The accumulation of GABA in inulin-primed lettuce following a *B. cinerea* infection is dependent on a functional ET signaling pathway (Tarkowski et al., 2019; Tarkowski et al., 2020). Taken together, these studies point to the possibility that fructans prime plant responses to fungal infections.

Recently, the potential of VOCs, which are emitted by plants in response to abiotic and biotic stresses (Picazo-Aragonés et al., 2020), as eco-friendly priming compounds has been proposed (Brilli et al., 2019). Especially, GLVs representing a group of plant VOCs have been shown to prime plant defense responses involving JA- and SA-regulated signaling (Engelberth et al., 2004; Li et al., 2016; Ameye et al., 2018). However, the exact mechanisms underlying this priming process remain to be elucidated. In addition, GLVs seem to have a rather direct effect on plant defense responses. Therefore, priming by GLVs is not reviewed in detail here.

Besides, high-throughput screening revealed that several other (structurally different) artificial compounds are capable to prime defense responses to *Pst* in *Arabidopsis* plants (Noutoshi et al., 2012a; Noutoshi et al., 2012b).

## 5 Concluding remarks

### 5.1 Common features of chemical priming

Priming of defense responses has been established as an integral part of IR in plants (De Kesel et al., 2021; Vlot et al., 2021). Priming in IR may happen without direct responses if the stimulus is weak, but it may also work in parallel with the direct responses if the stimulus is strong enough to induce a direct response. Primed and direct defense response vary significantly in their costs regarding plant growth and seed production. For example, whereas priming by BABA was associated with an only marginal decrease in growth, induction of direct defense responses by high concentrations of BABA or BTH caused a much stronger decrease in plant growth and even decreased the seed production (van Hulten et al., 2006). This indicates that under disease pressure, chemical priming positively influences the fitness of plants, while under conditions without a pathogen attack no significant differences in the plants’ fitness were detected. This is in contrast to induction of direct defense responses, which affects the plants’ fitness (van Hulten et al., 2006). This observation highlights the potential of chemical priming as a valuable tool for enhancing plant protection without negative growth-defense trade-offs. Besides primed/direct responses, IR results from either the local or the systemic establishment of plant defense responses allowing for the description of different IR phenotypes (De Kesel et al., 2021).

As described in this review, the exogenous application of various natural and artificial chemicals can enhance the disease resistance of plants. However, the ratio between primed and direct responses after chemical application varies, depending on the compounds itself and their concentrations. SA and its synthetic analog BTH are well-known for their ability to cause both direct and primed responses (Thulke and Conrath, 1998; Conrath, 2009). Also, NHP is able to directly induce SAR gene expression and concurently primes for enhanced defense activation (Hartmann and Zeier, 2019). In the case of BABA treatment, the concentration defines not only the establishment of primed/direct responses but also the spatial distribution of the plant responses (van Hulten et al., 2006; De Kesel et al., 2021). Intriguingly nearly all observed defense responses have been shown to be primed and systemically triggered in ISR by PGPR (De Kesel et al., 2021; Pieterse et al., 2021). This dualistic phenomenon is important in the context of potential applications. In summary, it requires the establishment of a ‘therapeutic window’ for each compound indicating an active concentration range that provides effective resistance enhancement with minimal adverse effects on fitness costs.

The molecular mechanisms underlying chemical priming have been at least partially resolved for several chemicals as graphically summarized in **Figures 1**-**5**. Treatment by chemical priming agents leads to various epigenetic and transcriptional changes, accumulation of receptors, inactive proteins and/or transcription factors as well as hormonal changes that can be observed prior to pathogen attacks. This primed state leads to earlier, faster and/or stronger defense responses and an enhanced resistance. The transcriptional coactivator and SA receptor NPR1 is the broadest regulator in chemical priming against pathogens. Unsurprisingly, activities of SA (Yi et al., 2014) and its synthetic analogue BTH (Kohler et al., 2002) are dependent on NPR1. Intriguingly also priming by MeJA and PGPR failed in *npr1* mutants (Pieterse et al., 1998). However, ISR triggered by PGPR is not dependent on SA itself (Pieterse et al., 2014). Also priming by NHP required the function of the transcriptional coregulator NPR1 (Chen et al., 2018; Hartmann et al., 2018; Yildiz et al., 2021). Despite that, NHP was shown to work also in an SA-independent manner (Bartsch et al., 2006; Mishina and Zeier, 2006; Bernsdorff et al., 2016). Additionally, priming by another mobile signal, AZA, was impaired in various SA synthesis and/or signaling mutants. BABA-IR to defend against biotic stresses involves priming of SA-dependent but also -independent defense responses (Cooper and Ton, 2022). As one more example, CK pretreatment did not potentiate pathogen resistance neither in the SA signaling mutant *npr1* nor in the SA-deficient mutant *eds16* (Choi et al., 2010; Argueso et al., 2012). Taken together, it is evident that NPR1 is a key regulator of priming with a wide range of compounds but not necessarily in an SA-dependent manner. Despite the crucial role or NPR1 during the priming of IR, the role of the transcriptional co-repressors NPR3/4 in priming still remains underexplored and is even neglected in recent studies.

Regulation of phytohormones and their signaling is another common feature of chemical priming. Positive regulation of SA biosynthesis was described for Pip/NHP (Chen et al., 2018; Hartmann et al., 2018; Yildiz et al., 2021), BABA (Pastor et al., 2014) and MeJA (Koley et al., 2022). Hx treatment, on the other hand, has negative effects on SA signaling (Scalschi et al., 2013) and depends on JA and ABA (Vicedo et al., 2009; Scalschi et al., 2013). As shown in **Table 1**, BABA treatment can affect SA, JA and ABA but also their combinations depending on the treated plant and pathogen. This observation was recently discussed for ISR triggered by PGPR in Vlot et al. (2021). It was proposed that the interplay of the different phytohormone signaling pathways determines the outcome of ISR and that this depends on the nature of the plant, the resistance inducer, and the phytopathogen (Vlot et al., 2021). Also, Koley et al. (2022) showed that SA and JA operate rather synergistically in priming against *Rhizoctonia solani* in tomato than following the ‘SA-JA antagonism’ rule. In the complex crosstalk of plant hormone signaling, it is unlikely for one hormone to work isolated from others, therefore the effect on various hormones should be always considered.

### 5.2 Chemical priming in agriculture

Compounds priming IR were initially hoped to be used as an alternative to traditional chemical biocides. However, due to their low efficiencies depending on the plant species and/or genotype and negative impacts on plant growth and yield, chemicals priming IR are not commonly used in agriculture. Also the ‘zero tolerance’ approach, meaning that the complete elimination of pests and pathogens was the central aim, did not leave much space for alternative techniques including chemical priming for IR in crop protection. As the complete elimination of pathogens is hardly ever achieved and the strong selection pressure that traditional chemical biocides execute on the surviving pathogens, the usage of those biocides risks the evolvement of resistant pathogens. In recent years, alternative strategies taking into account the improved understanding of plant-pathogen interactions are increasingly considered for plant protection. This offers new opportunities for chemical priming to be used in the field. Chemical priming agents have the advantage that they are (almost) not directly toxic to the environment or the pathogens themselves but still provide efficient protection of different plant species against various pathogens (Yassin et al., 2021). As an example, BTH application provided protection against a broad spectrum of diseases in a variety of crops in the field (Ryals et al., 1996). However, several parameters, such as concentrations, frequency of applications, the host genotype (Vallad and Goodman, 2004), and, in one case, the plants’ growth stage (Heil et al., 2000), influence the efficiency of BTH. In addition, it’s necessary to apply the compounds, such as BTH, in an preventive rather than curative manner, thereby limiting the economic success as farmers favor using standard curative fungicides (Conrath, 2009). Due to their above-mentioned potential negative impacts, chemical priming compounds will most likely be combined with other agents to enable protection against various stress conditions in the field (Yassin et al., 2021). Chemical priming agents were suggested as a part of integrated pest (crop) management (IPM), which is a coordinated and planned strategy for the environmentally sensitive prevention, detection and control of pests, weeds, and diseases (Yassin et al., 2021).

### 5.3 Future perspectives

Taken together, the compounds presented in the review highlight the broad potential of chemicals as plant priming agents to improve the defense against pathogen infections. Moreover, priming substances have been and will be very useful tools to study priming response in order to get further insight into the mechanisms and evolution of this fascinating process. Although structurally diverse compounds have the potential to prime plant defense responses against various pathogens, it seems that these induce in the end through similar signaling pathways. However, there is still partly only descriptive evidence for chemical priming to pathogen infections, pointing to the importance of investigating the molecular mechanisms further. Especially the observation that certain compounds convey resistance only to specific pathogens makes it important to fully investigate the underlying mechanisms and understand the reasons for specificity. For the majority of priming compounds, it also remains to be investigated how they are perceived by plants. In addition, it is not always clear how long chemicals prime pathogen defense responses or how the memory is conferred.

In view of the support of chemical priming for plants to defend successfully against various pathogens, chemical priming seems to be a promising tool for agricultural application despite the potential drawbacks mentioned above. In particular, priming by natural compounds may offer an opportunity to decrease the use of pesticides. Another interesting application would be the use of natural compounds for postharvest fruit preservation. However, so far none of the compounds has hold its promises in an agricultural context. In order to be effective and useful for application a priming compound would need not only be cheap in its production and non-toxic but also cause a reliable long-lasting improved stress resistance under diverse conditions. It is a future challenge to design screens and establish test systems to identify lead substances. Given the fact that apparently quite a number of structurally diverse chemicals is principally able to prime plant resistance the chemical space to be explored appears to be large. The accumulated knowledge on the plant immune system and gene response pattern to priming treatments may offer opportunities to identify novel priming substances.

## Author contributions

MH and VR wrote the draft of the manuscript. MH, VR, TS, and AC revised the manuscript. All authors contributed to the article and approved the submitted version.
